# The multifaceted mechanisms of Tanshinone IIA in doxorubicin-induced cardiotoxicity

**DOI:** 10.3389/fmed.2026.1783433

**Published:** 2026-03-25

**Authors:** Qinqin Wang, Danqi Zou, Huan Chen, Di Cui, Xupeng Huang, Yuqiu Chen, Yuxin Qi, Zheng Nan, Xiang Wang, Xiaoling Shang

**Affiliations:** 1 College of Traditional Chinese Medicine, Changchun University of Chinese Medicine, Changchun, China; 2 College of Basic Medicine, Changchun University of Chinese Medicine, Changchun, China; 3 The Affiliated Hospital of Changchun University of Chinese Medicine, Changchun, Jilin, China

**Keywords:** adriamycin, cardiac toxicity, drug delivery system, pharmacological mechanism of action, Tanshinone IIA

## Abstract

Doxorubicin (DOX)-induced cardiotoxicity significantly impairs patients' quality of life and long-term prognosis, and can lead to irreversible cardiac dysfunction and heart failure years or even decades after chemotherapy has ceased. Natural medicinal products represent a promising strategy for preventing or treating this cardiotoxicity, with *Salvia miltiorrhiza* (Danshen) demonstrating therapeutic properties in this context. Considerable attention has been focused on Tanshinone IIA, the principal lipophilic diterpenoid quinone compound found in Danshen, for its potential efficacy against doxorubicin-induced cardiac injury. Its therapeutic mechanisms include alleviating oxidative stress, inhibiting cardiomyocyte apoptosis, exerting anti-inflammatory effects in the myocardium, attenuating cardiac fibrosis, and modulating cardiomyocyte autophagy. This article provides an in-depth elaboration of the mechanisms and targets through which Tan IIA counteracts doxorubicin-induced cardiotoxicity. Furthermore, we explored Tan IIA drug delivery systems (DDS) and device-assisted delivery platforms, thus offering novel insights for future research and clinical translation of Tan IIA, as well as presenting new strategies for addressing doxorubicin-induced cardiac injury.

## 1 Introduction

Doxorubicin (DOX) is a highly effective antitumour agent that improves patient survival; however, it is concurrently associated with severe, dose-dependent cardiotoxicity, also known as doxorubicin-induced cardiomyopathy (DIC/AIC) (1–4). This toxicity manifests not only acutely during chemotherapy but also chronically, emerging years or even decades after treatment cessation as cardiomyopathy, leading to irreversible cardiac dysfunction and heart failure. Once a diagnosis of heart failure has been made, the 2-year survival rate is as low as 40% (5–8), which severely restricts the clinical utility of doxorubicin and compromises patients' long-term quality of life. Currently, dexrazoxane is the sole cardioprotective agent to have received FDA approval, and it is specifically indicated for patients receiving anthracycline-based chemotherapy. However, its application is limited by potential adverse effects, including myelosuppression, hepatotoxicity, and the development of secondary malignancies (9). Consequently, the development of effective and safe cardioprotective agents that mitigate chemotherapy-induced myocardial injury without compromising antitumor efficacy remains a critical and urgent challenge in the field of cardio-oncology (10, 11).

Tanshinone IIA (Tan IIA) is the predominant lipophilic diterpenoid quinone compound found in the traditional Chinese herb *Salvia miltiorrhiza* Bunge. The substance has attracted considerable attention on account of its multifarious pharmacological activities, which encompass antioxidant, anti-inflammatory, anti-fibrotic, anti-apoptotic, and anti-tumor effects, as well as cardioprotective properties (12–15). Chemotherapy-induced cardiotoxicity is a complex, multifactorial process involving numerous interrelated pathways, including oxidative stress driven by reactive oxygen species (ROS), mitochondrial dysfunction, cellular apoptosis, inflammatory responses, iron overload, and calcium dysregulation. In recent years, researchers have begun to explore the potential of Tan IIA in mitigating cardiotoxicity associated with chemotherapy, particularly damage induced by DOX. Preliminary *in vitro* and *in vivo* studies have demonstrated promising protective effects (16, 17). Evidence suggests that Tan IIA can increase the sensitivity of cancer cells to DOX chemotherapy. The observed synergy is achieved by the inhibition of the expression of multidrug resistance (MDR)-associated ABC transporters, such as P-gp, BCRP, and MRP1, thereby increasing the accumulation of DOX within the intracellular environment of tumor cells. In addition, Tan IIA has been observed to suppress ELTD1, thereby promoting vascular normalization and activating the JNK signaling pathway, thus potentiating the efficacy of PD-1 inhibitors. Collectively, these observations suggest a synergistic antitumor effect (18–21).

Significant efforts have notably been invested in developing novel drug delivery systems (DDS) and device-assisted administration strategies to enhance the targeting, solubility and bioavailability of Tan IIA, thereby achieving more efficient cardioprotection (22–24). While research has revealed Tan IIA's potential in treating various cancers, studies focusing on its protective effects against chemotherapy-induced cardiotoxicity and the underlying mechanisms are still in their infancy. This review aims to comprehensively summarize and update recent advances in the utilization of Tan IIA and its derivative, sodium tanshinone IIA sulfonate (STS), for attenuating doxorubicin (DOX)-induced cardiotoxicity. We will explore the molecular mechanisms underpinning their cardioprotective actions, examine innovative drug delivery approaches designed to enhance their efficacy, and seek to pinpoint precise pharmacological targets. Finally, current challenges and prospective research directions are discussed to broaden the therapeutic landscape of natural products in combating chemotherapy-associated cardiac damage.

## 2 Drug delivery systems and device-assisted administration systems for Tanshinone IIA

### 2.1 Drug delivery systems

A drug delivery system (DDS) can be defined as a device or method that efficiently transports a therapeutic agent to its target site, with the aim of optimizing the drug's absorption, distribution, metabolism, and excretion (ADME) profile. This approach has been demonstrated to enhance drug concentration at the desired location, reduce systemic toxicity, and improve patient compliance (25, 26). Intelligently responsive nanocarriers are considered to be a cutting-edge strategy in this field. These nanomachines have been engineered to exploit specific pathological microenvironmental cues, such as elevated ROS, low pH, or high enzymatic activity, to trigger precise drug release. This enables targeted therapy (27–31). The encapsulation of hydrophobic drugs within nanocarriers, including liposomes, micelles, and nanoparticles, has been demonstrated to enhance their dispersibility and stability in aqueous environments (32, 33). This improvement in stability has been shown to result in enhanced absorption, prolonged circulation time, and sustained therapeutic efficacy. It is evident that a number of delivery systems, specifically formulated for Tan IIA, have been developed to address DOX-induced cardiotoxicity. These delivery systems have exhibited considerable therapeutic potential.

#### 2.1.1 ROS responsive delivery systems

DOX-induced cardiotoxicity is closely associated with its excessive generation of ROS in cardiomyocytes (34). To address this core pathological mechanism, several smart drug delivery systems based on Tan IIA have been developed. These systems are designed to specifically target and respond to the high-ROS pathological microenvironment in the heart, enabling precise targeting and controlled release for optimal therapeutic efficacy. For instance, Zhao et al. constructed a ROS-responsive, mitochondria-targeted Tan IIA micelle (TK-TPP-TIIA@Ms) that utilizes a triphenylphosphonium (TPP) group to target ROS-rich mitochondria in cardiomyocytes and releases Tan IIA specifically in high-ROS environments. Experimental studies confirmed that this micelle effectively reduces DOX-induced ROS, inflammation, and apoptosis, significantly alleviating myocardial injury in heart failure mice (23).

Furthermore, a theranostic probe named TOP-B, constructed from a Tan IIA backbone, was developed to address AIC, which is closely linked to oxidative stress. As an H_2_O_2_-responsive prodrug, TOP-B allows for highly sensitive and selective ratiometric fluorescence tracking of H_2_O_2_ levels in AIC models and exhibits excellent mitochondrial targeting capability, providing a novel tool for integrated research and treatment of AIC (35). These studies demonstrate that ROS-responsive drug delivery systems enhance the protective effect of Tan IIA against DOX-induced cardiotoxicity (Tables 1, 2).

**Table 1:** The conveying system and improved features of Tan IIA.

**Delivery system**	**Improved properties**	**References**
TK-TPP-TIIA@Ms	Appropriate particle size, and zeta potential; and demonstrated good encapsulation efficiency, drug loading, and biological safety	(23)
TOP-B	Improve targeting and sensitivity	(35)
Tan IIA/CA	Enhance the solubility and stability of Tan IIA, increase the drug loading capacity, and achieve controlled drug release	(22)
MCVs	Enhance the synergistic effect of the drug combination and promote the protection of STS in Dox-induced cardiomyocyte apoptosis	(39)
R + T/Lipo/EXO	Improve the bioavailability, enhance targeting ability and sensitivity	(42)

**Table 2:** The research on the drug delivery system used for Tan IIA in AIC and other cardiovascular diseases.

**Delivery system**	**Disease**	**Experimental model**	**Targets**	**Main results**	**References**
TK-TPP-TIIA@Ms	DOX-induced HF	*In vivo*: C57/BL6 male mice;	Reduce IL-1β, IL-6, TNF-a, MDA; increase SOD and GSH levels	Reduced myocardial tissue damage caused by DOX, decreased cell apoptosis, lowered the expression of inflammatory factors, improved oxidative stress, improved cardiac function, and inhibited DOX-induced heart failure	(34)
		*In vitro*: H9c2 cells			
TOP-B	DIC	*In vivo*: male CS7BL/6 mice; Zebrafish;	NA	Early diagnosis and treatment of AIC	(35)
		*In vitro*: H9C2 cells			
Tan IIA/CA	Tumor	*In vitro*: CX 1 and HGC 27 cell	Inhibited cell proliferation and reduced the migration of HGC 27 and CX1 cells	Exhibits antitumor activity by promoting apoptosis, autophagy, ferroptosis, and immune effects	(22)
MCVs	Dox-induced cardiomyocyte apoptosis	*In vitro*: A549 cancer cells	Reduce Bax/Bcl-2, cleaved-caspase3	Reduced myocardial tissue damage caused by DOX, decreased cell apoptosis, lowered the expression of inflammatory factors, improved oxidative stress, improved cardiac function, and inhibited DOX-induced heart failure	(39)
R + T/Lipo/EXO	Blood poisoning	*In vivo*: male Bal b/c mice	Reduce TNF-a, IL-6 *In vitro*:H1299 (NCI-H1299) cells	Improving the inflammatory state of cells significantly enhanced the phagocytic ability of macrophages, reduced the expression of tumor necrosis factor-a, inhibited cell apoptosis, regulated the intestinal flora, and alleviated the phenomenon of immune suppression	(42)

AIC, anthracycline-induced cardiotoxicity; DOX, doxorubicin; HF, heart failure; STS, a soluble derivative of Tan IIA; IL-1β, interleukin-1β; IL-6, interleukin-6; TNF-α, tumor necrosis factor-α; SOD, superoxide dismutase; GSH, reduced glutathione; Bax/Bcl-, Bcl-2-associated X protein ratio/B-cell lymphoma 2; NA, not applicable.

#### 2.1.2 PH-responsive delivery systems

Given that the tumor microenvironment and ischemic myocardial regions typically exhibit a lower pH (36, 37), Ren et al. developed pH-responsive calcium alginate nanoparticles loaded with Tanshinone IIA (Tan IIA/CA) to enhance the solubility of Tan IIA and achieve controlled drug release. Although the primary focus of that study was on its anticancer mechanisms, such pH-responsive carriers are, in principle, also applicable for targeting areas of ischemic cardiac injury, offering a potential strategy for achieving localized high-concentration drug delivery to the heart (22) (Tables 1, 2).

#### 2.1.3 Collaborative co-delivery systems

In strategies for preventing and treating chemotherapy-induced cardiotoxicity, co-delivery systems encapsulate both chemotherapeutic agents and cardioprotective drugs within a single carrier, aiming to achieve synergistic therapeutic enhancement and toxicity reduction (38). For example, Zhang et al. developed multicompartment vesicles (MCVs) for the co-delivery of DOX and the water-soluble derivative STS. This system not only enhanced their synergistic antitumor effect on cancer cells but also significantly reduced DOX-induced cardiomyocyte apoptosis through carrier-mediated protection. This approach ingeniously leveraged the cardioprotective properties of STS while circumventing its potential interference with DOX's therapeutic efficacy (39). Research by Li et al. further supports the potential of combining Tan IIA with DOX to achieve both synergistic anticancer activity and reduced cardiotoxicity (18, 40) (Tables 1, 2).

#### 2.1.4 Exosome-mediated delivery systems

Plant-derived extracellular vesicles (EVs) are considered promising drug delivery carriers due to their favorable biocompatibility and low immunogenicity (41). Wu et al. constructed a tumor cell-derived exosome-hybridized nanosystem loaded with Tan IIA [denoted as (R + T/Lipo/EXO)]. Although this study focused on sepsis, it demonstrated that the system significantly enhances macrophage uptake and effectively suppresses inflammation and apoptosis (42). Such a biomimetic delivery strategy, utilizing surface modification with cell membranes or exosomes, can improve the targeting ability and cellular internalization efficiency of carriers (43). In the future, it may be worthwhile to explore the use of cardiomyocyte- or endothelial cell-derived exosomes to precisely deliver Tan IIA to injured myocardial tissue, potentially opening new avenues for more targeted protection against chemotherapy-induced cardiotoxicity (Tables 1, 2).

### 2.2 Device-assisted drug delivery systems

In addition to drug delivery systems, device-assisted administration approaches have also been employed to enhance the therapeutic efficacy of Tan IIA. Given that treatment options for anthracycline-induced cardiotoxicity (AIC) remain relatively limited, transdermal delivery offers a viable strategy for certain cardiovascular conditions by circumventing hepatic first-pass metabolism (44). Notably, Gu et al. (45) significantly improved the transdermal penetration of Tan IIA from patch formulations by loading the compound onto nanocrystals and porous silica. This technique provides a conceptual foundation for the non-invasive administration of Tan IIA.

Moreover, chitosan-based microneedle transdermal delivery systems have attracted attention due to their painless application and high absorption efficiency (46). In the context of myocardial infarction (MI) therapy, Fan et al. (47) developed an injectable biomaterial by dispersing Tan IIA with liquid metal particles in sodium alginate for intrapericardial administration. This localized approach enabled sustained drug release and prolonged retention within cardiac tissue, leading to significantly improved cardiac function. Such a localized, sustained-release strategy holds significant translational relevance for the prevention and treatment of chronic myocardial injury in AIC, as it ensures the prolonged maintenance of a therapeutic concentration of the protective agent in myocardial tissue.

## 3 The mechanism of action of Tan IIA on DOX-induced cardiotoxicity

### 3.1 Antioxidant stress

Oxidative stress is widely recognized as a primary mechanism underlying DOX-induced cardiomyopathy (48, 49). DOX has been demonstrated to generate excessive ROS through multiple pathways, resulting in lipid peroxidation and depletion of antioxidant enzymes. This process subsequently triggers apoptosis, necrosis, or senescence in cardiomyocytes, endothelial cells, and progenitor cells (5, 34). Furthermore, Evidence indicates that DOX directly impairs the function of intrinsic antioxidant enzymes, thereby exacerbating the oxidative stress burden (50). A mounting body of evidence suggests that the activation of nuclear factor erythroid 2–related factor 2 (Nrf2), a pivotal regulator of the cellular antioxidant defense system, plays a pivotal protective role in counteracting DOX-induced myocardial oxidative stress (50, 51).

The study by Guo et al. clearly demonstrated that Tan IIA mitigates DOX-induced oxidative stress and myocardial injury by promoting the nuclear accumulation of Nrf2 and activating its downstream antioxidant response element (ARE) signaling pathway. This action enhances the activity of antioxidant enzymes, including superoxide dismutase (SOD), catalase (CAT), and glutathione (GSH), reduces ROS generation, improves cell viability, and significantly restores DOX-impaired cellular morphology. Nrf2 knockdown experiments confirmed that suppressing Nrf2 expression reverses the increased cell survival rate conferred by Tan IIA pretreatment, indicating that the cardioprotective effect of Tan IIA against DOX-induced toxicity is mediated through the activation of the Nrf2 signaling pathway (34). Yarmohammadi et al. also highlighted the potential of Tan IIA to counteract DOX cardiotoxicity via the Nrf2/ARE pathway (50). Furthermore, Zhang et al. (52) found that Tan IIA protects cells from oxidative damage by modulating the Nrf2/NLRP3 signaling pathway, which reduces the production of ROS and malondialdehyde (MDA) while promoting the expression of SOD and CAT. Additional research has shown that pretreatment with Tan IIA alleviates DOX-induced myocardial damage in C57BL/6 mice by improving histopathology, lowering serum levels of cardiac enzymes, and enhancing the activity of antioxidant enzymes (53, 54).

**Figure 1:**
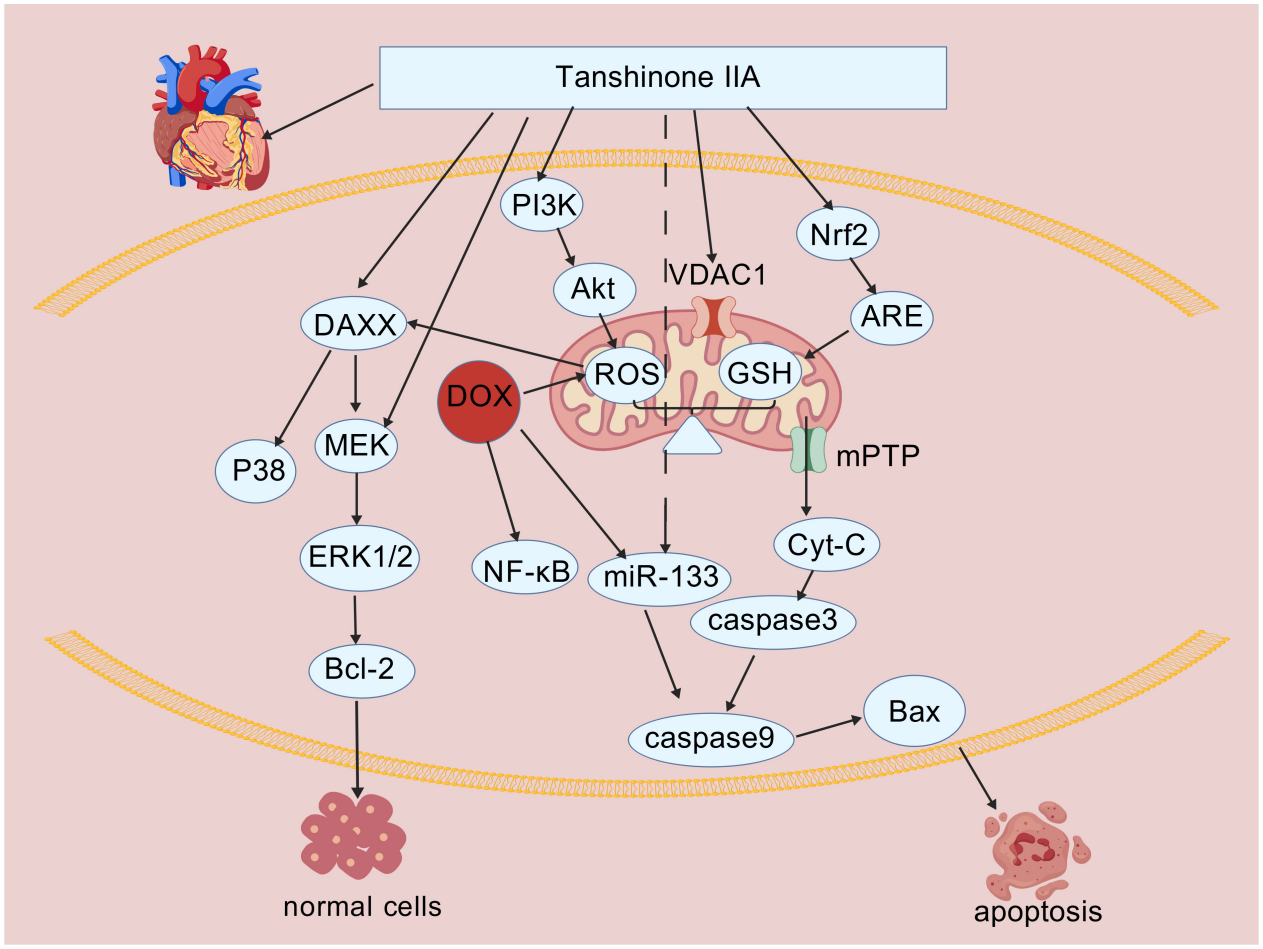
The protective mechanisms of Tan IIA against oxidative stress and cell apoptosis caused by DOX. Figure was created with BioGDP.com (135).

In summary, existing evidence indicates that Tan IIA primarily enhances myocardial antioxidant defense mechanisms by activating the Nrf2/ARE signaling pathway and its synergistic networks, thereby effectively counteracting DOX-induced oxidative stress-mediated cardiac damage. The specific mechanisms underlying its antioxidant effects are illustrated in Figure 1 (Table 3).

**Table 3:** The overview of the target/way/mechanism and effect of Tan IIA on AIC and other cardiovascular diseases.

**Models**	**Animals or cells**	**Range of dosage**	**The specific molecular mechanisms**	**Adjustment indicators**	**References**
AIC	H9c2 cells	1, 3, 5, 10 μM	Nrf2	Increased cell survival rate, significantly restored morphological changes, reduced ROS production, and elevated GSH levels	(34)
	Mice	15, 30 mg/kg			
Renal fibrosis caused by uric acid	HK-2cells	1, 2, 5, 10, 20, 50, 100 μM	Nrf2/NLRP3	Reduced the generation of ROS and MDA, promoted the expression of SOD and CAT, and significantly decreased the expression of TGF-β, FN and COL-1	(52)
AIC	Neonatal rat cardiomyocytes	0.5–2 μM/L	NA	Significantly reduced ROS production and prevented the decrease in the Bcl-2/Bax ratio caused by doxorubicin.	(68)
AIC	H9C2 cells	0.5–20 μM	Beclin1/LAMP1	Reduced the levels of LC3-II and P62, decreased the content of autolysosomes, increased the activity of tissue protease B; weakened the inhibitory phosphorylation of ULK1, increased the level of Beclin1 and the content of autophagosomes, inhibited mTOR activity, promoted the nuclear localization of TFEB, and activated the expression of LAMP	(53)
	Zebrafish	20 μM			
	Mice	10 mg/kg			
AIC	H9C2 and HL-1 cells	40 μM/L	DAXX/MEK/ERK1/2	Enhance cell vitality; induce the expression of DAXX, p-ERK1/2 and p-MEK, restore cardiac function, prevent changes in myocardial structure and myofibril destruction. Inhibit the expression of cleaved caspase-8, p-P38 and cleaved caspase-3 to suppress cell apoptosis	(54)
	C57BL/6 mice	2.5, 5 or 10 mg/kg			
AIC	Neonatal rat cardiomyocytes	0.1, 0.3, 1, 3 μM	Akt-signaling pathways	Enhance the phosphorylation of Akt in myocardial cells, inhibit the generation of ROS, reduce the quantity of cleaved caspase-3 and cytoplasmic cytochrome c, and increase the expression of Bcl-xL, thereby inhibiting myocardial cell apoptosis	(17)
AIC	H9c2 cells	NA	miR-133/caspase-9	Restoring miR-133 and inhibiting the Caspase-9 signaling cascade relieved myocardial apoptosis	(65)
Ethanol-induced myocardial apoptosis	C57BL/6 mice	5/10 mg/kg	PI3K/Akt	Down-regulate the expression of PDCD4 and activate the PI3K/Akt pathway to inhibit apoptosis	(59)
AIC	BALB/c nude mice	10 mg/kg	ERK1/2	Inhibit the activation of ERK1/2 in Dox-treated breast cancer cells, reduce the expression of c-Myc, increase the expression of caspase-3, and decrease the expression of MMP-2; promote the activation of ERK1/2 in Dox-treated cardiomyocytes, increase the expression of c-Myc, and decrease the expression of caspase-3	(40)
Myocardial ischemia	SD Rat	10 mg/kg	ERS and mitochondrial apoptosis signaling pathway	Reduced the apoptosis of myocardial tissue and the expression levels of caspase-3, Cyto c and Apaf-1 in myocardial tissue. Increased the survival rate of damaged myocardial cells, enhanced the expression of Bcl-2 and Bak, increased Bim and CHOP, decreased the production of TBARS, ROS and H_2_O_2_, reduced the expression of ATF4 and IRE1α, and decreased intracellular calcium and oxidative stress in myocardial cells	(66)
AIC	SD Rat	4.16 ml/kg DHI	NA	Significantly increased the expression level of Bcl-2, and prevented the increase in the expression of Bax and caspase-3	(67)
	H9c2 cells	10 μl/ml DHI			
IRI	H9c2 cells	8 μM	VDAC1	Significantly reduced cell survival rate, GPX4 and GSH expression, and the GSH/GSG ratio, and inhibited the upregulation of LDH activity, COX-2 and VDAC1 protein expression, ROS level, mitochondrial damage, and A/R-induced GSSG. Increased MMP and Bcl-2/Bax ratio, activated Caspase-3 and closed m PTP	(72)
AIC	H9c2 cardiomyocytes	0, 1.062, 2.125, 4.25, 8.5, 17, 34, 68 μM	NA	Reducing ROS expression, decreasing the secretion of inflammatory factors, restoring mitochondrial membrane potential, improving oxidative stress, alleviating myocardial injury, inhibiting apoptosis, and preventing heart failure	(23)
	C57/BL6 male mice	1.5 mg/kg			
HF induced by AMI	H9C2 cells	0.05 μg/ml, 0.1 μg/ml	TLR4/NF-κB p65	Reduce the levels of NT-pro-BNP, IL-1β and IL-18, and inhibit cardiomyocyte apoptosis. downregulate the expressions of inflammatory cell apoptosis-related proteins such as TLR4, NF-κB p65, IL-1β, NLRP3, Caspase-1 and GSDMD-N. Enhance the survival ability of cardiomyocytes, inhibit apoptosis, and prevent the nuclear translocation of NF-κB p65 protein	(16)
	Rat	1.5 mg/kg			
The inflammatory response of VSMCs induced by LPS	VSMC cells	25, 50, 100μM/L	TLR4/TAK1/NF-κB	Inhibit the expression of MCP-1, IL-6 and TNF-α, inhibit the production of NO, and suppress the expression of TLR4, the phosphorylation of TAK1, and the nuclear translocation of NF-κB (p65)	(76)
Atherosclerosis	HUVECs	2.5 μM, 5 μM, 10 μM	circ_0000231/miR-590-5p/TXNIP/NF-κB	Regulate the miR-590-5p/TXNIP axis to down-regulate circ_0000231 and activate the NF-κB signaling pathway	(77)
Atherosclerosis	ApoE^−/−^mice/HUVECs	10/20 mg/kg	AMPK	Inhibited the formation of NLRP3, ASC and GSDMD; inhibited the expression of IL-1β and IL-18; reduced the number of PI-positive cells, decreased the expression of mature caspase-1 and cleaved GSDMD, and lowered the level of activated IL-1β. Restored mitochondrial membrane potential and reduced the generation of mt ROS, and increased AMPK expression	(80)
Atherosclerosis	ApoE^−/−^mice	10 mg/kg	TGF-β/PI3K/Akt/eNOS	Increase the level of HDL-C, reduce the levels of TC, triglycerides and LDL-C; increase the level of nitric oxide and the ratio of nitric oxide to ET-1, reduce the levels of TNF-α, IL-6 and ET-1; upregulate the mRNA levels of TGF-β and PI3K. Downregulate the mRNA expressions of MMP-9, VEGF and HIF1-α. Increase the expression of HDL-C, and reduce the expressions of testosterone acid, triglycerides and LDL-C	(81)
HF induced by excessive stress	Rat	5, 10, 20 mg/kg	NA	Increase LVEF and LVFS, decrease LVID d, LVIDs, LVEDP, and LVSP; reduce HWI and LVWI	(89)
HF induced by AMI	SD Rat	1.5 mg/kg	Nox4	Reduce the expression levels of collagen I, collagen III, TGF-β, α-SMA, MMP2 and MMP9, increase the activity of superoxide dismutase and glutaldehyde, and decrease the activity of superoxide anion and NADPH oxidase	(90)
AIC	H9c2 cells	0, 1.6, 8, 40 μM	NA	Increase cell survival rate and improve cell apoptosis, alleviate the disorder of cardiac muscle cells, hydropic vacuolation and myofibril loss. Improve heart rate, ST interval, QRS wave group, myocardial contractility and myocardial tension	(84)
	Mice	NA			
Diabetic cardiomyopathy	Mice	10/50 mg/kg	ERS/SIRT1	It alleviated the pathological changes in the hearts of diabetic mice, improved the pathological morphology of myocardial cells, reduced the cell death rate, and inhibited the expression of ERS-related proteins and mRNAs	(104)

AIC, anthracycline-induced cardiotoxicity; IRI, ischemia-Reperfusion Injury; HF, heart failure; AMI, acute myocardial infarction; VSMCs, vascular smooth muscle cells; LPS, lipopolysaccharide; EPCs, endothelial progenitor cells; TNF-α, tumor necrosis factor-α; HUVECs, Human umbilical vein endothelial cells; CFs, cardiac fibroblasts; Nrf2, Nuclear factor (erythroid-derived 2)-like 2; ROS, reactive oxygen species; GSH, reduced glutathione; MDA, malondialdehyde; CAT, catalase; TGF-β, transforming growth factor-β; FN, fibronectin; COL-1, collagen type I; Bcl-2/Bax, B-cell lymphoma 2/Bcl-2-associated X protein ratio; LC3-II, microtubule-associated protein 1A/1B-light chain 3-phosphatidylethanolamine conjugate; P62, Sequestosome 1; ULK1, Unc-51 like autophagy activating kinase 1; mTOR, mammalian target of rapamycin; TFEB, transcription factor EB; LAMP, lysosome-associated membrane protein; DAXX, death domain-associated protein; p-ERK1/2, phosphorylated extracellular signal-regulated kinase 1/2; p-MEK, phosphorylated mitogen-activated protein kinase; p-P38, phosphorylated P38; Akt; ak strain transforming; miR-133, microRNA-133; PDCD4, programmed cell death 4; PI3K, phosphatidylinositol 3-kinase; ERK1/2, extracellular signal-regulated kinase 1/2; c-Myc, cellular Myc; MMP-2, matrix metalloproteinase-2; Apaf-1, apoptotic protease-activating factor-1; Cyt-c, cytochrome c; Bim, Bcl-2 interacting mediator of cell death; LAMP1, lysosomal-associated membrane protein 1; CHOP, C/EBP homologous protein; TBARS, thiobarbituric acid reactive substances; ATF4, activating transcription factor 4; IRE1α, inositol-requiring enzyme 1 alpha; Bax, Bcl-2-associated X protein; LDH, lactate dehydrogenase; COX-2, prostaglandin-endoperoxide synthase 2; VDAC1, voltage-dependent anion channel 1; GSSG, oxidized glutathione; MMP, matrix metallo proteinase; mPTP, mitochondrial permeability transition pore; NT-pro-BNP, N-terminal pro-brain natriuretic peptide; IL-18, interleukin-18; TLR4, toll-like receptor 4; NF-κB p65, nuclear factor kappa-light-chain-enhancer of activated B cells p65; IL-1β, interleukin-1β; NLRP3, NLR family pyrin domain containing 3; GSDMD-N, gasdermin D N-terminal domain; MCP-1, monocyte chemoattractant protein-1; IL-6, interleukin-6; NO, nitric oxide; MNCs, mononuclear cells; VCAM-1, vascular cell adhesion molecule-1; ICAM-1, intercellular adhesion molecule-1; NF-κB, nuclear factor kappa-light-chain-enhancer of activated B cells; IκB-α, nuclear factor of kappa light polypeptide gene enhancer in B-cells inhibitor-α; IKKα/β, inhibitor of nuclear factor kappa-B kinase subunit α/β; miR-590-5p/TXNIP, microRNA-590-5p/thioredoxin-interacting protein axis; circ_0000231, circular RNA 0000231; ASC, apoptosis-associated speck-like protein containing a CARD; GSDMD, gasdermin D; PI, propidium iodide; mtROS, mitochondrial reactive oxygen species; AMPK, adenosine monophosphate-activated protein kinase; HDL-C, high-density lipoprotein cholesterol; TC, total cholesterol; LD-C, low-density lipoprotein cholesterol; ET-1, endothelin-1; mRNA, messenger RNA; MMP-9, matrix metalloproteinase-9; VEGF, vascular endothelial growth factor; HIF1-α, hypoxia-inducible factor 1-alpha; sCD40L, soluble CD40 ligand; LVEF, left ventricular ejection fraction; LVFS, left ventricular fractional shortening; LVIDd, left ventricular internal diameter end-diastole; LVIDs, left ventricular internal diameter end-systole; LVEDP, left ventricular end-diastolic pressure; LVSP, left ventricular systolic pressure; HWI, heart weight index; LVWI, left ventricular weight index; α-SMA, α-smooth muscle actin; MMP9, matrix metalloproteinase-9; TIMP, tissue inhibitor of metalloproteinase; miR-618, microRNA-618; E.S, endoplasmic reticulum stress; NOX4, NADPH oxidase 4; SOD, superoxide dismutase; SD Ra: Sprague–Dawley Rat; DHI: Danhong injection; NA, not applicable.

### 3.2 Inhibition of cardiac cell apoptosis

#### 3.2.1 Regulation of the PI3K/Akt and ERK1/2 signaling pathways

Apoptosis constitutes a pivotal pathological basis for cardiomyocyte damage and heart failure in DOX-induced cardiotoxicity, a phenomenon that is strongly associated with the increased generation of ROS triggered by the drug (55, 56). Research by Fu et al. has demonstrated that Tan IIA confers cardioprotection by shielding cardiomyocytes from oxidative stress-induced apoptosis. This protective effect is achieved through mechanisms such as enhancing free radical scavenging and preventing lipid peroxidation (57).

Akt, a serine/threonine kinase, has been identified as a primary mediator of the downstream effects of PI3K, coordinating diverse intracellular signals and regulating cell proliferation and survival. As demonstrated in previous studies, the activation of the PI3K/Akt signaling pathway has been shown to protect myocardial tissue and prevent cardiomyocyte apoptosis (58). In a study by Hong and colleagues, Tan IIA was demonstrated to protect cardiomyocytes from DOX-induced apoptosis by enhancing Akt phosphorylation, inhibiting DOX-induced ROS production, reducing the release of cleaved Caspase-3 and cytochrome c, and increasing the expression of the anti-apoptotic protein Bcl-xL. Treatment with a PI3K inhibitor has been demonstrated to attenuate this protective effect. These results indicate that Tan IIA's ability to mitigate doxorubicin cardiotoxicity is associated with modulation of the PI3K/Akt pathway (17). This observation was subsequently corroborated by Deng et al. (59).

The ERK1/2 pathway has been demonstrated to play a significant role in DOX-induced cardiomyocyte apoptosis (60). Research by Li et al. (40) further revealed that Tan IIA activates the ERK1/2 pathway in cardiomyocytes, thereby alleviating DOX cardiotoxicity, whereas it inhibits the same pathway in breast cancer cells to enhance the chemotherapeutic efficacy of DOX. The findings demonstrate the dual function of Tan IIA as both a cardioprotective agent and a chemosensitizer. Xu et al. (54) further refined this mechanism, demonstrating that Tan IIA suppresses cardiomyocyte apoptosis and improves cardiac function by activating the DAXX/MEK/ERK1/2 signaling pathway.

In conclusion, Tan IIA counteracts DOX-induced cardiotoxicity by activating pro-survival signaling pathways such as PI3K/Akt and ERK1/2, thereby exerting an anti-apoptotic effect. It is particularly noteworthy that Tan IIA exerts a tissue-specific, bidirectional regulatory effect on the ERK1/2 pathway. The provision of cardioprotection and chemosensitization in a single agent represents a novel strategy to address the fundamental challenge of balancing efficacy and safety in cancer chemotherapy. This positions Tan IIA as a promising candidate for development into an ideal chemotherapeutic adjuvant with considerable potential for clinical application.

#### 3.2.2 Regulation of mitochondria and apoptotic proteins

Mitochondria are the most extensively and severely damaged organelles in DOX-induced cardiotoxicity (61). DOX directly impairs mitochondria, causing dose-dependent opening of the mitochondrial permeability transition pore and the release of molecules such as cytochrome c, which activates the caspase cascade and initiates cell necrosis or apoptosis (62, 63). Additionally, DOX disrupts the mitochondrial electron transport chain, impairing its function, reducing ATP production, and further promoting reactive oxygen species (ROS) generation (60, 64). Moreover, DOX-mediated ROS production increases the activity of cathepsin B, which subsequently mediates the release of apoptosis-inducing factor (AIF) from mitochondria through interaction with Bax aggregates. This process triggers extensive DNA damage, upregulates p53 expression, and activates poly (ADP-ribose) polymerase 1, ultimately leading to apoptotic cell death (60). This cascade results in a systematic reduction of cardiomyocytes and contributes to the development of cardiomyopathy. Collectively, these findings highlight that mitochondrial dysfunction and apoptotic proteins are critical mediators in DOX-induced cardiomyocyte injury.

In relation to the aforementioned mechanisms, numerous studies have shown that Tan IIA protects cardiomyocytes by inhibiting the mitochondrial apoptotic pathway at multiple points. It reduces the release of cytochrome c from mitochondria into the cytosol, upregulates the expression of anti-apoptotic proteins such as Bcl-2 and Bcl-xL, and downregulates the levels of pro-apoptotic proteins such as caspase-3, caspase-9, and Bax. Furthermore, the ability of Tan IIA to attenuate doxorubicin-induced cardiomyocyte apoptosis exhibits a degree of dose-dependency (17, 65–68). Additionally, miR-133 plays a critical role in DOX-induced cardiomyocyte apoptosis by regulating caspase-9 expression, thereby blocking the initiation of the DOX-triggered apoptotic cascade. DOX significantly suppresses miR-133 expression in H9c2 cardiomyocytes, leading to increased apoptosis. Treatment with Tan IIA markedly reverses this downregulation of miR-133 under pathological conditions, which in turn inhibits the activation of caspase-9 and its associated apoptotic effectors, thereby alleviating DOX-induced cardiomyocyte apoptosis (65). Collectively, these studies establish the foundation for Tan IIA-mediated cardioprotection through anti-apoptotic pathways. The specific mechanism by which Tan IIA counteracts DOX-induced cardiomyocyte apoptosis is illustrated in Figure 1.

Mitochondria-dependent ferroptosis also plays a critical role in the progression of AIC (69, 70). Specifically, the accumulation of free iron within mitochondria and the consequent lipid peroxidation and oxidative stress trigger ferroptosis, recognized as a primary mechanism of DOX-induced cardiomyocyte death (71). Research by Hu et al. revealed that Tan IIA can directly interact with voltage-dependent anion channel 1 (VDAC1), thereby simultaneously inhibiting the mitochondria-mediated apoptotic pathway and ferroptosis (72). This dual action positions Tan IIA as a potential therapeutic agent capable of counteracting both cell death pathways. To further enhance the targeting and efficacy of Tan IIA, Zhao et al. developed a ROS-responsive micellar delivery system for Tan IIA. This targeted delivery system effectively reduces DOX-induced ROS production, inflammatory response, and apoptosis in cardiomyocytes, while also restoring the damaged mitochondrial membrane potential, directly validating the high efficiency and specificity of targeted Tan IIA delivery in regulating mitochondrial function and apoptotic pathways (23).

Taken together, the available evidence indicates that Tan IIA effectively counteracts DOX-induced cardiotoxicity through multitargeted interventions that ameliorate mitochondrial dysfunction. By simultaneously blocking two key cell death pathways—apoptosis and ferroptosis—Tan IIA offers a more comprehensive strategy for combating DOX-related cardiac injury. The identification of a clear mitochondrial target, VDAC1, provides a molecular basis for developing highly selective drugs. Furthermore, validated targeted delivery strategies establish a reliable pathway for the efficient and low-toxicity clinical translation of this agent (Table 3).

### 3.3 Anti-inflammatory and anti-fibrotic effects

#### 3.3.1 Inhibition of inflammatory signaling pathways

A major mechanism of DOX-induced cardiotoxicity involves excessive ROS generation, which elevates inflammatory chemokines and cytokines, thereby perpetuating inflammatory responses (73–75). The nuclear factor-κB (NF-κB) pathway, a central regulator of inflammation, represents a critical therapeutic target in this process. Studies show that Tan IIA can effectively mitigate associated myocardial injury by suppressing NF-κB activation through multiple molecular pathways.

Tan IIA directly inhibits the nuclear translocation and transcriptional activity of NF-κB, thereby downregulating downstream inflammatory factors and mitigating resultant damage in both endothelial cells and vascular smooth muscle cells (VSMCs) (76–78). In endothelial cells, this action suppresses NF-κB activation, reducing TNF-α-induced expression of VCAM-1 and ICAM-1 to produce anti-inflammatory effects (78). Evidence also indicates that Tan IIA modulates key upstream inflammatory pathways. It alleviates cardiomyocyte pyroptosis and inflammation by inhibiting the TLR4/NF-κB p65 pathway in acute myocardial infarction, while partially attenuating LPS-induced inflammation in vascular smooth muscle cells through the TLR4/TAK1/NF-κB axis (16, 76). Beyond these direct mechanisms, Tan IIA also regulates NF-κB indirectly through complex molecular networks. Chen et al. demonstrated that it suppresses this pathway via the circ_0000231/miR-590-5p/TXNIP axis, consequently reducing inflammatory responses induced by oxidized low-density lipoprotein (77).

In terms of cellular function, Tan IIA exhibits broad protective effects. It can reverse the inhibitory effects of TNF-α on the proliferation, migration, adhesion, and angiogenic capacity of endothelial progenitor cells, while also reducing the secretion of related inflammatory cytokines (79). Moreover, STS has been shown to improve mitochondrial function and suppress mitochondrial ROS production by activating AMPK phosphorylation, ultimately inhibiting NLRP3 inflammasome activation (80). Additional studies suggest that its protective effects may also involve the activation of the TGF-β/PI3K/Akt/eNOS pathway, which alleviates endothelial and inflammatory injury while lowering blood lipids and stabilizing plaques (81).

**Figure 2:**
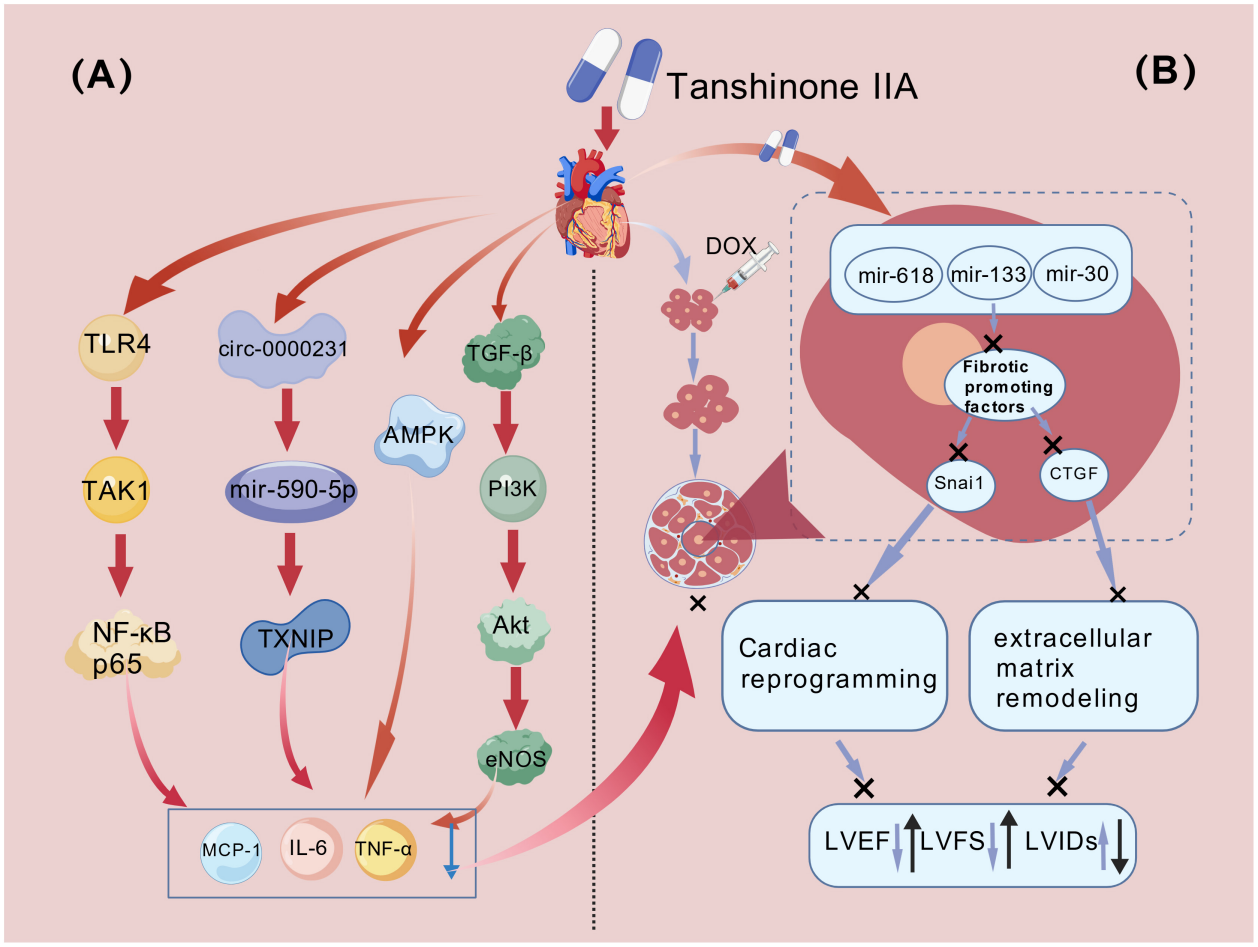
The protective mechanism of Danshenone IIA against doxorubicin-induced myocardial inflammation, fibrosis and remodeling. **(A)** Protective mechanism of Tan IIA against doxorubicin-induced myocardial inflammation and fibrosis. **(B)** Protective mechanism of Tan IIA against doxorubicin-induced myocardial fibrosis and cardiac remodeling. Figure was created with BioGDP.com (135).

In summary, inflammatory response is a central pathological event in DOX-induced cardiac injury. Through the multi-target synergistic actions described, Tan IIA effectively inhibits the NF-κB signaling pathway and its downstream inflammatory cascade, thereby providing a solid molecular pharmacological basis for mitigating secondary myocardial damage. The specific anti-inflammatory mechanisms are summarized in Figure 2A.

#### 3.3.2 Anti-fibrotic and cardiac remodeling effects

DOX can impair myocardial contractile and diastolic functions by triggering processes such as inflammation, fibrosis, vascular remodeling, and cardiomyocyte hypertrophy. These pathological changes lead to a variety of structural and functional cardiac abnormalities, ultimately resulting in heart failure and death (82). Experimental studies by Merten et al. have shown that cumulative doses of DOX induce hypertrophy in H9c2 rat cardiomyocytes (83). Research by Jiang et al. further confirmed that DOX-treated cells exhibit increased surface area and reduced cell number compared to normal H9c2 cells. In contrast, STS reverses DOX-induced cardiomyocyte hypertrophy, alleviates cellular disarray, reduces vacuolization and loss of myofibrils, and significantly improves cardiac contractility and myocardial tension (84).

Reportedly, miR-133 is involved in the regulation of myocyte proliferation and differentiation (85), and has been shown to improve cardiac reprogramming in mouse or human fibroblasts by directly targeting Snai1, a master regulator of epithelial-to-mesenchymal transition (86). Moreover, miR-30 and miR-133 directly regulate connective tissue growth factor (CTGF), playing an important role in myocardial extracellular matrix remodeling (87). In zebrafish experiments, Mishima et al. (88) found that miR-1 and miR-133 influence muscle gene expression and regulate the organization of sarcomeric actin. Experimental studies by Song et al. (65) demonstrated that Tan IIA ameliorates myocardial remodeling through regulation of miR-133 expression. Research by Wang et al. in zebrafish and C57BL/6 mice also found that Tan IIA ameliorates DOX-induced cardiac dysfunction and reverses structural abnormalities such as disorderly arrangement of cardiac tissue, myofibrillar loss, pyknosis, and plasma-dissolved cardiomyocytes (53). Another study demonstrated that Tan IIA dose-dependently reverses changes in left ventricular ejection fraction (LVEF), left ventricular fractional shortening (LVFS), and left ventricular internal dimension at end-systole (LVIDs) in DOX-treated groups, and significantly prevents DOX-induced structural alterations in cardiomyocytes including myocardial fiber breakage and nuclear pyknosis (54).

Furthermore, Li et al. showed that Tan IIA alleviates ventricular remodeling in pressure overload-induced heart failure rats by suppressing myocardial inflammatory factors (IL-6, CRP) and cardiomyocyte apoptosis (89). In a myocardial infarction model, Tan IIA reversed cardiac dysfunction and fibrosis through inhibition of oxidative stress mediated by Nox4 (90). Additionally, Jiang et al. revealed that Tan IIA exerts antifibrotic effects on cardiac fibroblasts and rat cardiac tissue by upregulating miR-618, thereby suppressing profibrotic factors (84). These findings collectively reinforce the protective role of Tan IIA against myocardial fibrosis and cardiac remodeling.

The collective evidence demonstrates that Tan IIA concurrently targets multiple pathological pathways, including inflammation, fibrosis, and cardiomyocyte hypertrophy. This multi-faceted action provides a comprehensive therapeutic strategy to counteract the complex myocardial remodeling induced by DOX. The key regulatory molecules involved establish well-defined molecular targets for developing precise cardioprotective agents. The specific mechanisms responsible for its anti-fibrotic and anti-remodeling effects are delineated in Figure 2B. These findings hold significant implications for improving the clinical management of DOX-induced chronic cardiomyopathy (Table 3).

### 3.4 Modulating autophagy and restoring autophagic flux

The cardiotoxicity induced by DOX is closely associated with its disruption of the autophagic process in cardiomyocytes. Autophagy, a critical mechanism for maintaining cellular homeostasis, metabolic balance, organelle function, and redox stability (63, 91–93), shows a significant link between its dysregulation and cardiovascular diseases, particularly anthracycline-induced cardiotoxicity (AIC) and the resulting heart failure (53, 92, 94). DOX impairs cellular homeostasis and contributes to cardiotoxicity by disrupting the dynamic balance between autophagosomes and autolysosomes and downregulating autophagic flux in cardiomyocytes such as H9c2 cells (53).

The complete autophagic process relies on the coordinated action of multiple sequential stages. During the initiation phase, the UNC-51-like kinase 1 (ULK1) complex drives the synthesis of the double-membrane autophagosome. The multiprotein complex assembled by the autophagy-related protein Beclin 1 with Vps34 and UVRAG is subsequently phosphorylated and activated by ULK1, thereby mediating autophagosome formation (95). The maturation phase is characterized by the lipidation of microtubule-associated protein 1 light chain 3 (LC3), marked by the conversion of the cytosolic form LC3-I to its membrane-bound counterpart, LC3-II (96). Finally, in the degradation stage, the autophagosome fuses with the lysosome to form an autolysosome, within which the encapsulated contents are degraded by lysosomal acid hydrolases, a process facilitated by lysosome-associated membrane proteins 1 and 2 (LAMP1/2) (97).

DOX disrupts autophagy by activating its core negative regulator, mTOR. Specifically, mTOR phosphorylates UNC-51-like kinase 1 (ULK1) at Ser757, inhibiting its activity. This indirectly inactivates Beclin1, thereby blocking autophagosome formation during the autophagy initiation phase (92). Concurrently, mTOR phosphorylates the basic helix-loop-helix leucine zipper transcription factor EB (TFEB), resulting in its cytoplasmic retention and impaired nuclear localization. Consequently, this suppresses the expression of TFEB target genes, including those involved in lysosomal biogenesis, such as lysosomal hydrolases, and lysosome-associated membrane protein 1 (LAMP1), which ultimately disrupts autophagic flux (98). Furthermore, DOX inhibits the activity of cathepsin B, impairing lysosomal proteolysis. This interference with autolysosomal degradation leads to the accumulation of autolysosomes, hinders autophagosome formation, and reduces overall autophagic flux.

Tan IIA corrects the abnormal autophagic flux induced by DOX through a dual mechanism involving enhanced autophagosome formation and autolysosomal degradation, thereby improving cardiomyocyte viability (53). In zebrafish and C57BL/6 mouse models of anthracycline-induced cardiotoxicity, Tan IIA's restoration of autophagic function via upregulation of Beclin1 and LAMP1 expression, as demonstrated by Wang et al., involves increased activity of cathepsin B, thereby improving lysosomal proteolysis. Furthermore, Tan IIA inhibits mTOR activation and downregulates phosphorylation of ULK1 at Ser757, which facilitates the recovery of nuclear TFEB levels. Collectively, these actions restore cardiomyocyte function, maintain structural collagen deposition, and reverse DOX-induced cardiotoxicity (53).

**Figure 3:**
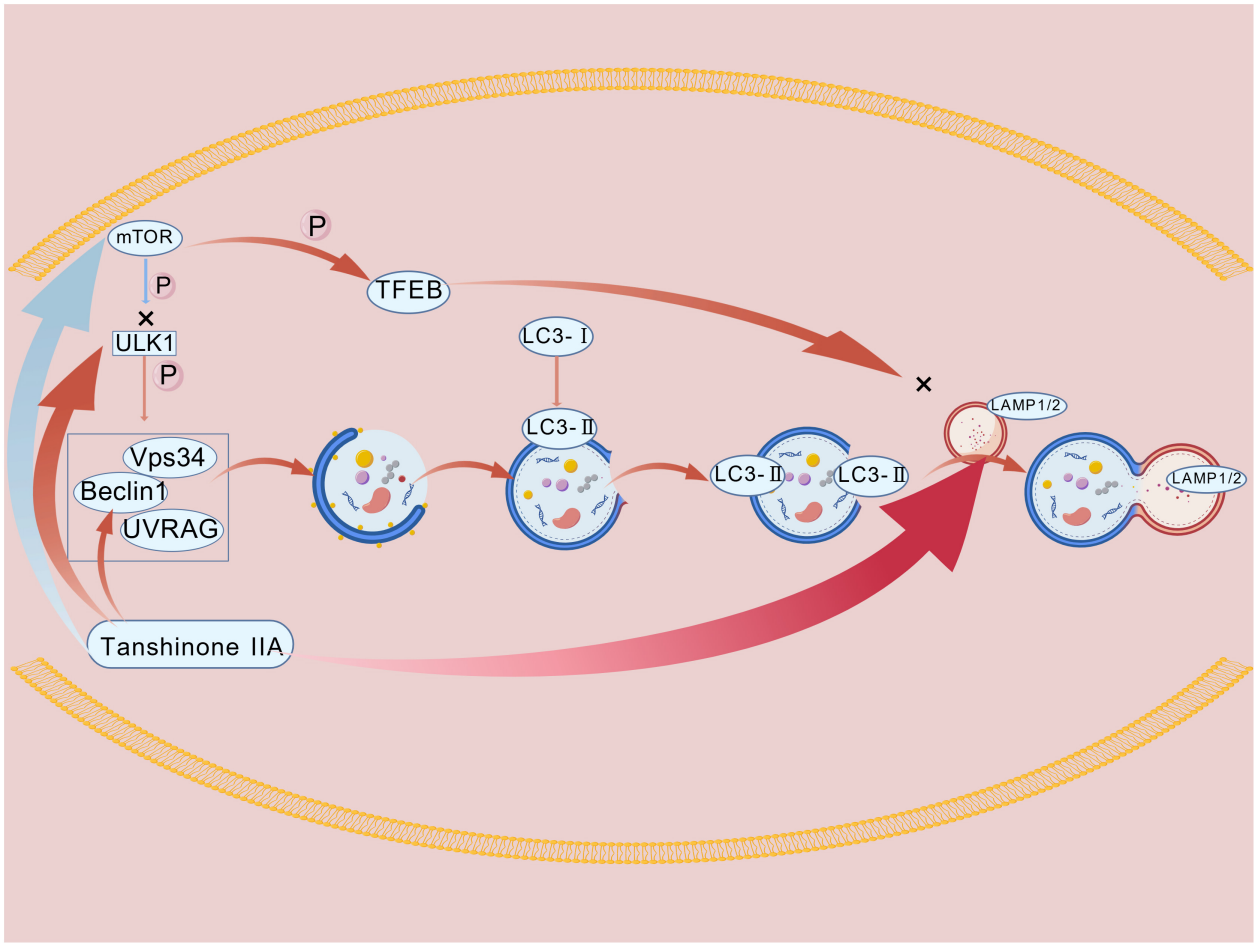
The protective mechanism of Tan IIA against the abnormal autophagic flux caused by DOX. Figure was created with BioGDP.com (135).

The studies discussed above together establish that Tan IIA mitigates DOX-induced cardiotoxicity via multi-target regulation of autophagic flux, with the specific mechanisms involved detailed in Figure 3. Through its dual regulatory actions on the synthesis, maturation, clearance, and lysosomal biogenesis phases of the autophagy process, Tan IIA provides a precise and comprehensive strategy for intervening in the autophagic imbalance caused by DOX. The key pathways identified, such as those involving Beclin1 and LAMP1, offer well-defined targets for developing novel cardioprotective agents centered on the modulation of autophagy (Table 3).

### 3.5 Other potential mechanisms

Tan IIA has also been shown to modulate other pathophysiological processes closely associated with cardiovascular diseases, including calcium ion homeostasis, cellular energy metabolism, endoplasmic reticulum stress, and systemic gut-brain axis regulation. In the context of DOX-induced cardiotoxicity, disruption of calcium homeostasis within cardiomyocytes represents a key injury mechanism (60, 99). Tan IIA may exert cardioprotective effects by regulating relevant calcium signaling pathways, a potential mechanism preliminarily observed in models of cardiotoxicity induced by sunitinib (100).

Metabolic dysfunction represents a key driver of doxorubicin-induced cardiotoxicity (101), and Tan IIA has been demonstrated to ameliorate myocardial energy metabolism. Specifically, Zhou et al. reported that Tan IIA activates the hypoxia-inducible factor 1α (HIF-1α) signaling pathway and upregulates glucose transporter 1 (GLUT-1) expression. This action may enhance glucose uptake and utilization in cardiomyocytes, thereby alleviating energy metabolism impairment (102).

Endoplasmic reticulum (ER) stress represents a pivotal mechanism in DOX-induced cardiotoxicity (103). Tan IIA mitigates excessive ER stress and maintains ER homeostasis, thereby reducing consequent cardiomyocyte apoptosis and dysfunction, partly through upregulating deacetylase SIRT1. This protective mechanism has also been validated in other models of ER stress-related cardiomyopathies (104).

Furthermore, the cardioprotective properties of Tan IIA may extend to systemic regulatory networks (104). DOX can compromise intestinal barrier function during treatment, leading to low-grade endotoxemia that exacerbates cardiac inflammation and injury (105). Research by Zhu et al. demonstrated that Tan IIA improves the composition of the gut microbiota, reduces intestinal mucosal permeability and local inflammation, lowers circulating levels of lipopolysaccharide (LPS), and indirectly exerts cardioprotective effects through modulation of the gut-brain axis (106).

Collectively, these studies indicate that the protective effect of Tan IIA against DOX-induced cardiotoxicity stems from a multi-target, multi-pathway approach. This encompasses intracellular mechanisms including calcium homeostasis regulation, support for energy metabolism, and maintenance of endoplasmic reticulum stability, as well as systemic modulation of gut-brain axis function. By systematically alleviating the multi-faceted cardiac injury caused by DOX, this evidence provides a robust pharmacological rationale for its potential clinical application.

## 4 Clinical applications of Tanshinone IIA in doxorubicin-induced cardiotoxicity

In the field of adjuvant cancer therapy, pharmaceutical preparations containing Tan IIA are widely used to mitigate DOX-induced cardiotoxicity. Network pharmacology studies and experimental research indicate that Danshen Injection and Danhong Injection can counteract acute myocardial injury through multi-target mechanisms (67, 107). Specifically, Yi et al. demonstrated that Danhong Injection alleviated DOX-induced cardiotoxicity in rats by inhibiting apoptotic pathways, as evidenced by improvements in electrocardiographic parameters and reductions in serum biochemical markers (67). Furthermore, a clinical study by Kong et al. showed that the combined use of Tongluo Ningxin Formula with anthracycline-based chemotherapy significantly reduced the incidence of ST-T segment abnormalities, decreased QRS wave voltage, and lowered the occurrence of arrhythmias in patients. It improved cardiac function, as reflected by an increase in left ventricular ejection fraction (LVEF), and modulated serum markers of myocardial status, elevating cardiac troponin T (cTnT) and superoxide dismutase (SOD) levels (108).

Tan IIA, particularly in its sulfonated form, has demonstrated clinical benefits in cardiovascular contexts. Research by Peng et al. and Wu et al. indicates that STS, when combined with standard therapy, reduces the frequency and duration of angina episodes, improves electrocardiographic findings and clinical symptoms, and lowers levels of blood lipids and inflammatory markers (109, 110). The findings from a randomized controlled trial by Li et al. offered further support for the anti-inflammatory effect of adjunctive STS in coronary heart disease, manifested as significant reductions in systemic markers including hs-CRP and cellular inflammatory cytokines (111). Collectively, this evidence establishes the clinical efficacy of STS in ameliorating myocardial ischemia and inflammation, two key pathogenic pathways that strongly overlap with the mechanisms underlying DOX-related cardiotoxicity.

More importantly, Tan IIA may bring additional benefits to chemotherapy patients with underlying cardiovascular diseases; this drug has both antihypertensive effects and AIC effects, serving as a highly promising intervention for cancer patients with comorbid hypertension, demonstrating significant potential for synergistic treatment of multiple diseases, and providing a new therapeutic option for special cancer populations (112). Although Tan IIA has shown important value in the field of cancer comorbidities, direct clinical studies on special populations such as elderly cancer patients and those with comorbid diabetes mellitus, hyperlipidemia, or potential cardiovascular diseases remain relatively scarce at present, and its synergistic advantages in synchronously regulating underlying diseases during cancer treatment have not yet been fully verified and explored, which makes in-depth exploration in this direction have important clinical significance and research prospects. Therefore, future studies should focus on the efficacy, safety, and mechanism of action of Tan IIA in cancer patients with comorbidities such as metabolic diseases and cardiovascular diseases, clarify its application value in special populations, and provide high-quality evidence-based medical basis for promoting clinical translation and formulating individualized treatment strategies.

As a commonly used cardioprotective drug in clinical practice, Dexrazoxane has a definite effect in preventing doxorubicin-related cardiotoxicity while Tanshinone IIA shows superior potential value in some cardioprotective effects. A meta-analysis systematically evaluated the role of various drugs in the prevention and treatment of anthracycline-induced cardiotoxicity (AIC), and the results demonstrated that Dexrazoxane and Tan IIA have their own advantages in improving different cardiac function indicators. Specifically, both compound Salvia miltiorrhiza preparations containing Tan IIA and Qili Qiangxin Capsules are more effective than Dexrazoxane in improving left ventricular ejection fraction (LVEF), while Dexrazoxane is slightly more effective than *Salvia miltiorrhiza* preparations in reducing the level of myocardial enzyme CK-MB after chemotherapy (113). These study results confirm that Tan IIA has important research significance in the field of anthracycline-induced cardiotoxicity, and we therefore compared the two drugs in terms of efficacy, safety, and action targets (Table 4). At present, clinical studies directly comparing the two are still relatively limited, so future research needs not only to deepen the clinical exploration of Tan IIA but also to strengthen head-to-head comparison and combined application research of the two drugs, thereby providing more direct reference evidence for the rational clinical application of Tan IIA.

**Table 4:** The advantages and disadvantages of dexrazoxane and Tanshinone IIA in terms of differences in efficacy, safety profile, and therapeutic targets.

**Drug characteristics**	**Dexrazoxane**	**Tanshinone IIA**
Core target	It has a single target and strong specificity, mainly reducing the formation of doxorubicin-iron complexes through iron chelation to inhibit oxidative stress, inhibiting topoisomerase IIβ to prevent DNA damage, and suppressing the p38 MAPK/NF-κB pathway to reduce cardiomyocyte apoptosis and necrosis (123–126)	It targets a variety of molecules and acts through a wide range of signaling pathways. It mainly exerts potent antioxidant effects by activating the Nrf2 pathway, exerts anti-inflammatory effects by inhibiting the NF-κB pathway, maintains cardiomyocyte homeostasis by regulating the autophagy pathway, and protects myocardial mitochondrial function while attenuating myocardial fibrosis and ventricular remodeling, thereby reducing cardiomyocyte apoptosis (17, 50, 51, 53, 54, 65–68, 77)
Therapeutic characteristics	It possesses a high level of evidence but has limitations in clinical application. In the FDA-approved indication (metastatic breast cancer with a cumulative doxorubicin dose >300 mg/m^2^), it can significantly reduce the risk of heart failure by approximately 80%, whereas data for other tumor types and pediatric patients remain limited (9, 127)	It has a wide range of effects, but the level of evidence needs to be improved. In addition to cardioprotection, it has auxiliary therapeutic value for various cardiovascular diseases and can provide additional benefits for patients with comorbid underlying diseases; however, there is currently a lack of direct, large-sample RCT evidence to prove that it can reduce the incidence of heart failure in the population, and the clinical improvement effect on key indicators such as LVEF also needs to be confirmed by higher-quality studies (112, 128–130)
Safety profile	This drug is associated with well-defined adverse reactions, and caution is warranted when administered in combination with other agents. The primary adverse effect is the potential induction of secondary malignancies, including acute myeloid leukemia and myelodysplastic syndrome, which may increase the burden of chemotherapy resistance. Additional adverse reactions include nausea, alopecia, mucositis, vomiting, and elevated liver enzymes (9, 131–133)	Adverse reactions are mild with good tolerability. Hypersensitivity reactions are the main clinical manifestations, such as rash and phlebitis, and fever, abdominal pain are rare (134)

MAPK, mitogen-activated protein kinase; NF-κB, nuclear factor kappa-light-chain-enhancer of activated B cells; Nrf2, nuclear factor (erythroid-derived 2)-like 2.

## 5 Challenges and future prospects

Tan IIA demonstrates significant potential in preventing and treating DOX-induced cardiotoxicity; however, its clinical translation faces multiple challenges. The inherent lipophilicity of Tan IIA leads to poor aqueous solubility and low oral bioavailability, which represents a primary barrier to its widespread application (24). Although its sulfonated derivative, STS, addresses the solubility issue, its stability, targeting specificity, and clearance rate within the body require further optimization. Furthermore, high concentrations of Tan IIA or STS have shown potential cytotoxicity or developmental toxicity in certain models such as zebrafish embryos, HEK293 cells, and vascular smooth muscle cells (VSMCs) (114, 115), necessitating precise control over dosage and targeted release.

Additionally, the dual regulatory mechanism of Tan IIA, which enhances chemosensitivity in tumor cells while protecting cardiomyocytes, necessitates a high degree of *in vivo* targeting specificity (40). Consequently, the development of intelligent dual-targeting delivery systems capable of distinguishing between the tumor microenvironment and the injured myocardial microenvironment is critical for achieving optimal therapeutic outcomes. Future progress in the clinical translation and application of Tan IIA in cardio-oncology will be significantly advanced through rational formulation design based on pharmacokinetic principles, precise investigations of drug-drug interactions, and exploration of alterations in its pharmacokinetic behavior under pathological conditions (116).

Current clinical evidence supporting the preventive and therapeutic effects of Tan IIA and its sulfonated derivative (STS) against DOX-induced cardiotoxicity predominantly derives from systematic evaluations of traditional Chinese medicinal compound formulations. A notable limitation remains the scarcity of high-quality randomized controlled clinical trials, which constrains the full realization of their clinical value (109, 117). Future research efforts should prioritize the development of more sophisticated intelligent targeted delivery systems to achieve ultra-precise targeting and controlled drug release within cardiomyocytes. Concurrently, continued exploration of localized administration strategies is essential to facilitate the long-term, sustained release and maintenance of high Tan IIA concentrations at cardiac sites, thereby effectively addressing the challenge of chronic cardiotoxicity (47, 118).

On the molecular level, AIC is closely linked to the non-specific inhibition of topoisomerase II beta (Top2β) (119, 120). Future investigations should aim to clarify whether Tan IIA influences the activity or degradation of Top2β, which would help determine if its cardioprotective mechanism parallels that of the standard protective agent dexrazoxane (55). Notably, research has revealed that Tan IIA inhibits ferroptosis, mediated through the voltage-dependent anion channel 1 (VDAC1), an important mechanism underlying its cardioprotective action (69, 72). Subsequent studies could systematically evaluate the cardioprotective efficacy of Tan IIA within the pathological context of iron metabolism dysregulation induced by DOX.

Furthermore, the regulatory role of Tan IIA in autophagy warrants more in-depth investigation specifically in cardiomyocytes (121). It is worth noting that the application of network pharmacology and artificial intelligence technologies holds promise for more effectively predicting the multi-target interaction networks of Tan IIA, optimizing compound formulation compatibility, accelerating the design of novel DDS, and enhancing pharmacokinetic/pharmacodynamic (PK/PD) predictions. This integrated approach can thereby provide robust guidance for preclinical research and formulation optimization (122).

## 6 Conclusions and perspective

Tan IIA, the primary lipophilic active constituent derived from the traditional Chinese herb *Salvia miltiorrhiza* (Danshen), demonstrates significant therapeutic potential in preventing and treating DOX-induced cardiotoxicity. Its core protective effects stem from multifaceted pharmacological actions, including potent antioxidant activity, inhibition of cardiomyocyte apoptosis, and attenuation of inflammatory responses and fibrosis. Substantial research confirms that Tan IIA and its derivatives effectively mitigate DOX-induced myocardial injury and improve cardiac function. Notably, Tan IIA possesses a dual capability: it acts as a chemosensitizer in tumor cells while providing protection to cardiomyocytes. This unique profile positions it as an ideal adjunctive strategy in chemotherapy. Importantly, novel drug delivery technologies, particularly ROS-responsive and mitochondria-targeted nanocarriers, as well as synergistic co-delivery systems, offer promising solutions to challenges such as its low bioavailability. These approaches hold potential for achieving precise and controlled drug release at the site of cardiac injury. Currently, STS injection has established a foundation for clinical application in cardiovascular diseases. However, the evidence level for Tan IIA in managing DOX cardiotoxicity requires further elevation through well-designed, high-standard prospective clinical trials. Future research should focus on elucidating the key molecular mechanisms underlying its cardioprotective effects, optimizing intelligent targeted delivery systems to enhance its pharmacokinetic profile, and conducting rigorous clinical studies. These efforts will ultimately facilitate the translation of Tan IIA into a widely adopted clinical therapy for AIC.

## References

[1] Volkova M Russell R. Anthracycline cardiotoxicity: prevalence, pathogenesis and treatment. Curr Cardiol Rev. (2011) 7:214–20. doi: 10.2174/157340311799960645 22758622 PMC3322439

[2] Allemani C Weir HK Carreira H Harewood R Spika D Wang X-S . Global surveillance of cancer survival 1995–2009: analysis of individual data for 25,676,887 patients from 279 population-based registries in 67 countries (CONCORD-2). Lancet. (2015) 385:977–1010. doi: 10.1016/S0140-6736(14)62038-9 25467588 PMC4588097

[3] Herrmann J. Adverse cardiac effects of cancer therapies: cardiotoxicity and arrhythmia. Nat Rev Cardiol. (2020) 17:474–502. doi: 10.1038/s41569-020-0348-1 32231332 PMC8782611

[4] Lipshultz SE Colan SD Gelber RD Perez-Atayde AR Sallan SE Sanders SP. Late cardiac effects of doxorubicin therapy for acute lymphoblastic leukemia in childhood. N Engl J Med. (1991) 324:808–15. doi: 10.1056/NEJM199103213241205 1997853

[5] Cappetta D Rossi F Piegari E Quaini F Berrino L Urbanek K . Doxorubicin targets multiple players: a new view of an old problem. Pharmacol Res. (2018) 127:4–14. doi: 10.1016/j.phrs.2017.03.016 28336372

[6] Otero FJ Boor PJ Sheahan RG. Doxorubicin-induced cardiomyopathy. Am J Med Sci. (2000) 320:59–63. 10910374

[7] Schirone L D'Ambrosio L Forte M Genovese R Schiavon S Spinosa G . Mitochondria and doxorubicin-induced cardiomyopathy: a complex interplay. Cells. (2022) 11:2000. doi: 10.3390/cells11132000 35805084 PMC9266202

[8] Yu J Wang C Kong Q Wu X Lu JJ Chen X. Recent progress in doxorubicin-induced cardiotoxicity and protective potential of natural products. Phytomed Int J Phytother Phytopharmacol. (2018) 40:125. doi: 10.1016/j.phymed.2018.01.009 29496165

[9] Monahan DS Flaherty E Hameed A Duffy GP. Resveratrol significantly improves cell survival in comparison to dexrazoxane and carvedilol in a h9c2 model of doxorubicin induced cardiotoxicity. Biomed Pharmacother. (2021) 140:111702. doi: 10.1016/j.biopha.2021.111702 34015579

[10] European Hematology Association (EHA) the the European Society for Therapeutic Radiology and Oncology (ESTRO) and the International Cardio-Oncology Society (IC-OS): developed by the task force on cardio-oncology of the European Society of Cardiology (ESC). Eur Heart J Cardiovasc Imaging (2023) 24:e98. doi: 10.1093/ehjci/jead080 37076917

[11] Bernstein D. Response by Bernstein to letter regarding article. “Anthracycline cardiotoxicity: worrisome enough to have you quaking?” *Circ Res*. (2018) 122:e64–5. doi: 10.1161/CIRCRESAHA.118.312921 PMC5997269 29599280

[12] Guo R Li L Su J Li S Duncan SE Liu Z . Pharmacological activity and mechanism of Tanshinone IIA in related diseases. Drug Des Dev Ther. (2020) 14:4735–48. doi: 10.2147/DDDT.S266911 33192051 PMC7653026

[13] Du L Guan C Zhang H Jia H Wan Q. Harnessing the therapeutic value of Tanshinone IIA: a breakthrough therapy in cardiovascular diseases. Front Pharmacol. (2025) 16:1620152. doi: 10.3389/fphar.2025.1620152 40689206 PMC12270896

[14] Lu TC Wu YH Chen WY Hung YC. Targeting oxidative stress and endothelial dysfunction using Tanshinone IIA for the treatment of tissue inflammation and fibrosis. Oxid Med Cell Longev. (2022) 2022:2811789. doi: 10.1155/2022/2811789 35432718 PMC9010204

[15] Dabbaghi MM Soleimani Roudi H Safaei R Baradaran Rahimi V Fadaei MR Askari VR. Unveiling the mechanism of protective effects of Tanshinone as a new fighter against cardiovascular diseases: a systematic review. Cardiovasc Toxicol. (2024) 24:1467. doi: 10.1007/s12012-024-09921-x 39306819

[16] Chai R Ye Z Xue W Shi S Wei Y Hu Y . Tanshinone IIA inhibits cardiomyocyte pyroptosis through TLR4/NF-κB p65 pathway after acute myocardial infarction. Front Cell Dev Biol. (2023) 11:1252942. doi: 10.3389/fcell.2023.1252942 37766966 PMC10520722

[17] Hong H-J Liu J-C Chen P-Y Chen J-J Chan P Cheng T-H. Tanshinone IIA prevents doxorubicin-induced cardiomyocyte apoptosis through Akt-dependent pathway. Int J Cardiol. (2012) 157:174–9. doi: 10.1016/j.ijcard.2010.12.012 21190747

[18] Li K Liu W Zhao Q Wu C Fan C Lai H . Combination of Tanshinone IIA and doxorubicin possesses synergism and attenuation effects on doxorubicin in the treatment of breast cancer. Phytother Res. (2019) 33:1658–69. doi: 10.1002/ptr.6353 30945389

[19] Mao D Wang H Guo H Che X Chen M Li X . Tanshinone IIA normalized hepatocellular carcinoma vessels and enhanced PD-1 inhibitor efficacy by inhibiting ELTD1. Phytomedicine. (2024) 123:155191. doi: 10.1016/j.phymed.2023.155191 38000104

[20] Li K Lai H. TanshinoneIIA enhances the chemosensitivity of breast cancer cells to doxorubicin through down-regulating the expression of MDR-related ABC transporters. Biomed Pharmacother. (2017) 96:371–7. doi: 10.1016/j.biopha.2017.10.016 29028589

[21] Zhang Y-Z Lai H-L Huang C Jiang Z-B Yan H-X Wang X-R . Tanshinone IIA induces ER stress and JNK activation to inhibit tumor growth and enhance anti-PD-1 immunotherapy in non-small cell lung cancer. Phytomedicine. (2024) 128:155431. doi: 10.1016/j.phymed.2024.155431 38537440

[22] Ren T Wang J Ma Y Huang Y Yoon S Mu L . Preparation of pH-responsive Tanshinone IIA-loaded calcium alginate nanoparticles and their anticancer mechanisms. Pharmaceutics. (2025) 17:66. doi: 10.3390/pharmaceutics17010066 39861714 PMC11768977

[23] Zhao Y Wang J Zhang Z Kong L Liu M Chen M . A ROS-responsive TPP-modified Tanshinone IIA micelle improves DOX-induced heart failure. Int J Pharm. (2025) 672:125318. doi: 10.1016/j.ijpharm.2025.125318 39921016

[24] Zou Y Zheng Y Liu Y Zhao H Zhang Y Zhao Q . Supramolecular self-assembly of Tanshinone IIA to construct an antitumor drug delivery platform. ACS Omega. (2025) 10:35916–29. doi: 10.1021/acsomega.5c03030 40860716 PMC12371760

[25] Chien S-T Suydam IT Woodrow KA. Prodrug approaches for the development of a long-acting drug delivery systems. Adv Drug Deliv Rev. (2023) 198:114860. doi: 10.1016/j.addr.2023.114860 37160248 PMC10498988

[26] Osterberg L Blaschke T. Adherence to medication. N Engl J Med. (2005) 353:487–97. doi: 10.1056/NEJMra050100 16079372

[27] Zhang C Pan D Li J Hu J Bains A Guys N . Enzyme-responsive peptide dendrimer-gemcitabine conjugate as a controlled-release drug delivery vehicle with enhanced antitumor efficacy. Acta Biomater. (2017) 55:153. doi: 10.1016/j.actbio.2017.02.047 28259838

[28] Zhang Y Guo Q An S Lu Y Li J He X . ROS-switchable polymeric nanoplatform with stimuli-responsive release for active targeted drug delivery to breast cancer. ACS Appl Mater Interfaces (2017) 9:12227. doi: 10.1021/acsami.6b16815 28350451

[29] Du J-Z Du X-J Mao C-Q Wang J. Tailor-made dual pH-sensitive polymer-doxorubicin nanoparticles for efficient anticancer drug delivery. J Am Chem Soc. (2011) 133:17560–3. doi: 10.1021/ja207150n 21985458

[30] Deng X Liang S Cai X Huang S Cheng Z Shi Y . Yolk-shell structured Au Nanostar@metal-organic framework for synergistic chemo-photothermal therapy in the second near-infrared window. Nano Lett. (2019) 19:6772–80. doi: 10.1021/acs.nanolett.9b01716 31496257

[31] Nie W Wu G Zhang J Huang L-L Ding J Jiang A . Responsive exosome nano-bioconjugates for synergistic cancer therapy. Angew Chem Int Ed Engl. (2020) 59:2018–22. doi: 10.1002/anie.201912524 31746532

[32] None A Saraf S. Applications of novel drug delivery system for herbal formulations. Fitoterapia. (2010) 81:680. doi: 10.1016/j.fitote.2010.05.001 20471457

[33] Huang L Huang XH Yang X Hu JQ Zhu YZ Yan PY . Novel nano-drug delivery system for natural products and their application. Pharmacol Res (2024) 201:107100. doi: 10.1016/j.phrs.2024.107100 38341055

[34] Guo Z Yan M Chen L Fang P Li Z Wan Z . Nrf2-dependent antioxidant response mediated the protective effect of Tanshinone IIA on doxorubicin-induced cardiotoxicity. Exp Ther Med. (2018) 16:3333. doi: 10.3892/etm.2018.6614 30233680 PMC6143869

[35] Cao Z Li J Yang W Cui J Xiang P Li Y . Harnessing the power of a H_2_O_2_-activated theranostic probe against doxorubicin-induced cardiotoxicity. ACS Sens. (2025) 10:5999–6009. doi: 10.1021/acssensors.5c01445 40726332

[36] Stubbs M McSheehy PM Griffiths JR Bashford CL. Causes and consequences of tumour acidity and implications for treatment. Mol Med Today. (2000) 6:15. doi: 10.1016/S1357-4310(99)01615-9 10637570

[37] Zou J Zhang F Zhang S Pollack SF Elsabahy M Fan J . Poly(ethylene oxide)-block-polyphosphoester-graft-paclitaxel conjugates with acid-labile linkages as a pH-sensitive and functional nanoscopic platform for paclitaxel delivery. Adv Healthc Mater. (2014) 3:441. doi: 10.1002/adhm.201300235 23997013 PMC3938983

[38] Al Bostami RD Abuwatfa WH Husseini GA. Recent advances in nanoparticle-based co-delivery systems for cancer therapy. Nanomaterials (Basel). (2022) 12:2672. doi: 10.3390/nano12152672 35957103 PMC9370272

[39] Zhang X Zong W Cheng W Han X. Codelivery of doxorubicin and sodium Tanshinone IIA sulfonate using multicompartmentalized vesosomes to enhance synergism and prevent doxorubicin-induced cardiomyocyte apoptosis. J Mater Chem B. (2018) 6:5243–7. doi: 10.1039/C8TB01136B 32254761

[40] Li S FeiyuTeng Zhang J Zhang P Li M Wang X . Tanshinone IIA potentiates the chemotherapeutic effect of doxorubicin against breast cancer cells and attenuates the cardiotoxicity of doxorubicin by regulating ERK1/2 pathway. J Biochem Mol Toxicol. (2024) 38:e23851. doi: 10.1002/jbt.23851 39267350

[41] Lian MQ Chng WH Liang J Yeo HQ Lee CK Belaid M . Plant-derived extracellular vesicles: recent advancements and current challenges on their use for biomedical applications. J Extracell vesicles (2022) 11:e12283. doi: 10.1002/jev2.12283 36519808 PMC9753580

[42] Wu Q Dong Q-Q Wang S-H Lu Y Shi Y Xu X-L . Tumor cell-derived exosomal hybrid nanosystems loaded with rhubarbic acid and Tanshinone IIA for sepsis treatment. J Inflamm Res. (2024) 17:5093–112. doi: 10.2147/JIR.S457978 39099664 PMC11296366

[43] Zhang S Zheng B Wei Y Liu Y Yang L Qiu Y . Bioinspired ginsenoside Rg3 PLGA nanoparticles coated with tumor-derived microvesicles to improve chemotherapy efficacy and alleviate toxicity. Biomater Sci. (2024) 12:2672–88. doi: 10.1039/D4BM00159A 38596867

[44] Carter P Narasimhan B Wang Q. Biocompatible nanoparticles and vesicular systems in transdermal drug delivery for various skin diseases. Int J Pharm. (2019) 555:49–62. doi: 10.1016/j.ijpharm.2018.11.032 30448309

[45] Gu X Guo J Mai Y Niu Y Chen J Zhao Q . Improved transdermal permeability of Tanshinone IIA from cataplasms by loading onto nanocrystals and porous silica. Pharm Dev Technol. (2021) 26:1061–72. doi: 10.1080/10837450.2021.1980800 34511025

[46] Li H Cui J Zhang T Lin F Zhang G Feng Z. Research progress on chitosan microneedle arrays in transdermal drug delivery. Int J Nanomed. (2024) 19:12957. doi: 10.2147/IJN.S487313 39651356 PMC11624690

[47] Fan L Qu H Wang B Li H-Z Yang W-W Guo H . Delivery of liquid metal particles and Tanshinone IIA into the pericardial cavity for myocardial infarction treatment. J Mater Chem B. (2024) 12:11916–25. doi: 10.1039/D4TB01274G 39445792

[48] Stěrba M Popelová O Vávrová A Jirkovský E Kovaríková P Geršl V . Oxidative stress, redox signaling, and metal chelation in anthracycline cardiotoxicity and pharmacological cardioprotection. Antioxid Redox Signal. (2013) 18:899–929. doi: 10.1089/ars.2012.4795 22794198 PMC3557437

[49] Octavia Y Tocchetti CG Gabrielson KL Janssens S Crijns HJ Moens AL. Doxorubicin-induced cardiomyopathy: from molecular mechanisms to therapeutic strategies. J Mol Cell Cardiol. (2012) 52:1213–25. doi: 10.1016/j.yjmcc.2012.03.006 22465037

[50] Yarmohammadi F Rezaee R Karimi G. Natural compounds against doxorubicin-induced cardiotoxicity: a review on the involvement of Nrf2/ARE signaling pathway. Phytother Res. (2021) 35:1163–75. doi: 10.1002/ptr.6882 32985744

[51] Wang Y Liao J Luo Y Li M Su X Yu B . Berberine alleviates doxorubicin-induced myocardial injury and fibrosis by eliminating oxidative stress and mitochondrial damage via promoting Nrf-2 pathway activation. Int J Mol Sci. (2023) 24:3257. doi: 10.3390/ijms24043257 36834687 PMC9966753

[52] Zhang W Feng J Liu R Xiang T Wu X. Tanshinone IIA regulates NRF2/NLRP3 signal pathway to restrain oxidative stress and inflammation in uric acid-induced HK-2 fibrotic models. Endocr Metab Immune Disord Drug Targets. (2025) 25:721–31. doi: 10.2174/0118715303315786240926075342 39473254 PMC12376118

[53] Wang X Li C Wang Q Li W Guo D Zhang X . Tanshinone IIA restores dynamic balance of autophagosome/autolysosome in doxorubicin-induced cardiotoxicity via targeting Beclin1/LAMP1. Cancers (Basel). (2019) 11:910. doi: 10.3390/cancers11070910 31261758 PMC6679133

[54] Xu L He D Wu Y Shen L Wang Y Xu Y. Tanshinone IIA inhibits cardiomyocyte apoptosis and rescues cardiac function during doxorubicin-induced cardiotoxicity by activating the DAXX/MEK/ERK1/2 pathway. Phytomedicine. (2022) 107:154471. doi: 10.1016/j.phymed.2022.154471 36182795

[55] Popelová O Štěrba M Hašková P Šimunek T Hroch M Gunčová I . Dexrazoxane-afforded protection against chronic anthracycline cardiotoxicity *in vivo*: effective rescue of cardiomyocytes from apoptotic cell death. British journal of cancer (2009) 101:792. doi: 10.1038/sj.bjc.6605192 19623174 PMC2736842

[56] Zhao L Zhang B. Doxorubicin induces cardiotoxicity through upregulation of death receptors mediated apoptosis in cardiomyocytes. Sci Rep. (2017) 7:44735. doi: 10.1038/srep44735 28300219 PMC5353581

[57] Fu J Huang H Liu J Pi R Chen J Liu P. Tanshinone IIA protects cardiac myocytes against oxidative stress-triggered damage and apoptosis. Eur J Pharmacol. (2007) 568:213–21. doi: 10.1016/j.ejphar.2007.04.031 17537428

[58] Matsui T Tao J Del Monte F Lee KH Li L Picard M . Akt activation preserves cardiac function and prevents injury after transient cardiac ischemia *in vivo*. Circulation. (2001) 104:330. doi: 10.1161/01.CIR.104.3.330 11457753

[59] Deng H Yu B Li Y. Tanshinone IIA alleviates acute ethanol-induced myocardial apoptosis mainly through inhibiting the expression of PDCD4 and activating the PI3K/Akt pathway. Phytother Res. (2021) 35:4309–23. doi: 10.1002/ptr.7102 34169595

[60] Wenningmann N Knapp M Ande A Vaidya TR Ait-Oudhia S. Insights into doxorubicin-induced cardiotoxicity: molecular mechanisms, preventive strategies, and early monitoring. Mol Pharmacol. (2019) 96:219–32. doi: 10.1124/mol.119.115725 31164387

[61] Sarvazyan N. Visualization of doxorubicin-induced oxidative stress in isolated cardiac myocytes. Am J Physiol. (1996) 271:H2079–2085. doi: 10.1152/ajpheart.1996.271.5.H2079 8945928

[62] Wallace KB Sardão VA Oliveira PJ. Mitochondrial determinants of doxorubicin-induced cardiomyopathy. Circ Res. (2020) 126:926–41. doi: 10.1161/CIRCRESAHA.119.314681 32213135 PMC7121924

[63] Miyamoto S. Autophagy and cardiac aging. Cell Death Differ. (2019) 26:653–64. doi: 10.1038/s41418-019-0286-9 30692640 PMC6460392

[64] de Oliveira BL Niederer S. A biophysical systems approach to identifying the pathways of acute and chronic doxorubicin mitochondrial cardiotoxicity. PLoS Comput Biol. (2016) 12:e1005214. doi: 10.1371/journal.pcbi.1005214 27870850 PMC5117565

[65] Song T Yao Y Wang T Huang H Xia H. Tanshinone IIA ameliorates apoptosis of myocardiocytes by up-regulation of miR-133 and suppression of Caspase-9. Eur J Pharmacol. (2017) 815:343–50. doi: 10.1016/j.ejphar.2017.08.041 28867607

[66] Fang Y Duan C Chen S Liu Z Jiang B Ai W . Tanshinone-IIA inhibits myocardial infarct via decreasing of the mitochondrial apoptotic signaling pathway in myocardiocytes. Int J Mol Med. (2021) 48:158. doi: 10.3892/ijmm.2021.4991 34212981 PMC8262657

[67] Yi X Wang F Feng Y Zhu J Wu Y. Danhong injection attenuates doxorubicin-induced cardiotoxicity in rats via suppression of apoptosis: network pharmacology analysis and experimental validation. Front Pharmacol. (2022) 13:929302. doi: 10.3389/fphar.2022.929302 36071840 PMC9441549

[68] Gao J Yang G Pi R Li R Wang P Zhang H . Tanshinone IIA protects neonatal rat cardiomyocytes from adriamycin-induced apoptosis. Transl Res. (2008) 151:79–87. doi: 10.1016/j.trsl.2007.11.005 18201675

[69] Fang X Wang H Han D Xie E Yang X Wei J . Ferroptosis as a target for protection against cardiomyopathy. Proc Natl Acad Sci U S A. (2019) 116:2672. doi: 10.1073/pnas.1821022116 30692261 PMC6377499

[70] Tadokoro T Ikeda M Ide T Deguchi H Ikeda S Okabe K . Mitochondria-dependent ferroptosis plays a pivotal role in doxorubicin cardiotoxicity. JCI Insight (2020) 5:e132747. doi: 10.1172/jci.insight.132747 32376803 PMC7253028

[71] Xie L-H Fefelova N Pamarthi SH Gwathmey JK. Molecular mechanisms of ferroptosis and relevance to cardiovascular disease. Cells. (2022) 11:2726. doi: 10.3390/cells11172726 36078133 PMC9454912

[72] Hu T Zou H-X Le S-Y Wang Y-R Qiao Y-M Yuan Y . Tanshinone IIA confers protection against myocardial ischemia/reperfusion injury by inhibiting ferroptosis and apoptosis via VDAC1. Int J Mol Med. (2023) 52:109. doi: 10.3892/ijmm.2023.5312 37800609 PMC10558218

[73] Nagy A Börzsei D Hoffmann A Török S Veszelka M Almási N . A comprehensive overview on chemotherapy-induced cardiotoxicity: insights into the underlying inflammatory and oxidative mechanisms. Cardiovasc Drugs Ther. (2025) 39:1185. doi: 10.1007/s10557-024-07574-0 38492161 PMC12602570

[74] Ye S Su L Shan P Ye B Wu S Liang G . LCZ696 Attenuated doxorubicin-induced chronic cardiomyopathy through the TLR2-MyD88 complex formation. Front Cell Dev Biol. (2021) 9:654051. doi: 10.3389/fcell.2021.654051 33928085 PMC8076895

[75] Shi S Chen Y Luo Z Nie G Dai Y. Role of oxidative stress and inflammation-related signaling pathways in doxorubicin-induced cardiomyopathy. Cell Commun Signal CCS. (2023) 21:61. doi: 10.1186/s12964-023-01077-5 36918950 PMC10012797

[76] Meng Z Si C-Y Teng S Yu X-H Li H-Y. Tanshinone IIA inhibits lipopolysaccharide-induced inflammatory responses through the TLR4/TAK1/NF-κB signaling pathway in vascular smooth muscle cells. Int J Mol Med. (2019) 43:1847–58. doi: 10.3892/ijmm.2019.4100 30816448

[77] Chen Z Zhao J Wang S Li Q. Tanshinone IIA attenuates ox-LDL-induced endothelial cell injury by inhibiting NF-kapaB pathway via circ_0000231/miR-590-5p/TXNIP axis. Chem Biol Drug Des. (2024) 103:e14394. doi: 10.1111/cbdd.14394 37955049

[78] Yang J-X Pan Y-Y Ge J-H Chen B Mao W Qiu Y-G . Tanshinone II A attenuates TNF-α-induced expression of VCAM-1 and ICAM-1 in endothelial progenitor cells by blocking activation of NF-κB. Cell Physiol Biochem. (2016) 40:195–206. doi: 10.1159/000452537 27855363

[79] Wang X-X Yang J-X Pan Y-Y Zhang Y-F. Protective effects of Tanshinone IIA on endothelial progenitor cells injured by tumor necrosis factor-α. Mol Med Rep. (2015) 12:4055–62. doi: 10.3892/mmr.2015.3969 26095681 PMC4526031

[80] Zhu J Chen H Guo J Zha C Lu D. Sodium Tanshinone IIA sulfonate inhibits vascular endothelial cell pyroptosis via the AMPK signaling pathway in atherosclerosis. J Inflamm Res. (2022) 15:6293–306. doi: 10.2147/JIR.S386470 36408328 PMC9673812

[81] Wang J He X Chen W Zhang N Guo J Liu J . Tanshinone IIA protects mice against atherosclerotic injury by activating the TGF-β/PI3K/Akt/eNOS pathway. Coron Artery Dis. (2020) 31:385–92. doi: 10.1097/MCA.0000000000000835 31842027 PMC7192539

[82] Panpan T Yuchen D Xianyong S Meng L Ruijuan H Ranran D . Cardiac remodelling following cancer therapy: a review. Cardiovasc Toxicol. (2022) 22:771–86. doi: 10.1007/s12012-022-09762-6 35877038

[83] Merten KE Jiang Y Feng W Kang YJ. Calcineurin activation is not necessary for doxorubicin-induced hypertrophy in H9c2 embryonic rat cardiac cells: involvement of the phosphoinositide 3-kinase-Akt pathway. J Pharmacol Exp Ther. (2006) 319:934–40. doi: 10.1124/jpet.106.108845 16926266

[84] Jiang B Zhang L Wang Y Li M Wu W Guan S . Tanshinone IIA sodium sulfonate protects against cardiotoxicity induced by doxorubicin in vitro and in vivo. Food Chem Toxicol Int J Published Br Ind Biol Res Assoc. (2009) 47:1538. doi: 10.1016/j.fct.2009.03.038 19358873

[85] Feng Y Niu L-L Wei W Zhang W-Y Li X-Y Cao J-H . A feedback circuit between miR-133 and the ERK1/2 pathway involving an exquisite mechanism for regulating myoblast proliferation and differentiation. Cell Death Dis. (2013) 4:e934. doi: 10.1038/cddis.2013.462 24287695 PMC3847338

[86] Muraoka N Yamakawa H Miyamoto K Sadahiro T Umei T Isomi M . MiR-133 promotes cardiac reprogramming by directly repressing snai1 and silencing fibroblast signatures. EMBO J. (2014) 33:1565. doi: 10.15252/embj.201387605 24920580 PMC4198052

[87] Duisters RF Tijsen AJ Schroen B Leenders JJ Lentink V van der Made I . miR-133 and miR-30 regulate connective tissue growth factor: implications for a role of microRNAs in myocardial matrix remodeling. Circ. Res. (2009) 104:170. doi: 10.1161/CIRCRESAHA.108.182535 19096030

[88] Mishima Y Abreu-Goodger C Staton AA Stahlhut C Shou C Cheng C . Zebrafish miR-1 and miR-133 shape muscle gene expression and regulate sarcomeric actin organization. Genes Dev. (2009) 23:619. doi: 10.1101/gad.1760209 19240126 PMC2658521

[89] Li X Xiang D Shu Y Zeng X Li Y. Mitigating effect of Tanshinone IIA on ventricular remodeling in rats with pressure overload-induced heart failure. Acta Cir. Bras. (2019) 34:e201900807. doi: 10.1590/s0102-865020190080000007 31618407 PMC6802940

[90] Chen R Chen W Huang X Rui Q. Tanshinone IIA attenuates heart failure via inhibiting oxidative stress in myocardial infarction rats. Mol Med Rep. (2021) 23:404. doi: 10.3892/mmr.2021.12043 33786621 PMC8025468

[91] Nakai A Yamaguchi O Takeda T Higuchi Y Hikoso S Taniike M . The role of autophagy in cardiomyocytes in the basal state and in response to hemodynamic stress. Nat Med. (2007) 13:619–24. doi: 10.1038/nm1574 17450150

[92] Orogo AM Gustafsson ÅB. Therapeutic targeting of autophagy: potential and concerns in treating cardiovascular disease. Circ. Res. (2015) 116:489. doi: 10.1161/CIRCRESAHA.116.303791 25634972 PMC4313578

[93] Gurusamy N Lekli I Gherghiceanu M Popescu LM Das DK. BAG-1 induces autophagy for cardiac cell survival. Autophagy. (2009) 5:120–1. doi: 10.4161/auto.5.1.7303 19001866

[94] Tannous P Zhu H Johnstone JL Shelton JM Rajasekaran NS Benjamin IJ . Autophagy is an adaptive response in desmin-related cardiomyopathy. Proc Natl Acad Sci U S A. (2008) 105:9745–50. doi: 10.1073/pnas.0706802105 18621691 PMC2474535

[95] Russell RC Tian Y Yuan H Park HW Chang Y-Y Kim J . ULK1 induces autophagy by phosphorylating Beclin-1 and activating VPS34 lipid kinase. Nat Cell Biol. (2013) 15:741–50. doi: 10.1038/ncb2757 23685627 PMC3885611

[96] Nath S Dancourt J Shteyn V Puente G Fong WM Nag S . Lipidation of the LC3/GABARAP family of autophagy proteins relies on a membrane-curvature-sensing domain in Atg3. Nat Cell Biol. (2014) 16:415–24. doi: 10.1038/ncb2940 24747438 PMC4111135

[97] Bartlett JJ Trivedi PC Pulinilkunnil T. Autophagic dysregulation in doxorubicin cardiomyopathy. J Mol Cell Cardiol. (2017) 104:1–8. doi: 10.1016/j.yjmcc.2017.01.007 28108310

[98] Moruno-Manchon JF Uzor N-E Kesler SR Wefel JS Townley DM Nagaraja AS . TFEB ameliorates the impairment of the autophagy-lysosome pathway in neurons induced by doxorubicin. Aging. (2016) 8:3507–19. doi: 10.18632/aging.101144 27992857 PMC5270683

[99] Tscheschner H Meinhardt E Schlegel P Jungmann A Lehmann LH Müller OJ . CaMKII activation participates in doxorubicin cardiotoxicity and is attenuated by moderate GRP78 overexpression. PLoS ONE. (2019) 14:e0215992. doi: 10.1371/journal.pone.0215992 31034488 PMC6488194

[100] Huang X Cui G Wang L Lee SM. Sodium Tanshinone IIA sulfonate prevents Sunitinib-induced cardiotoxicity in H9c2 cells in vitro and zebrafish in vivo. Pharmacol Clin Chin Mater Med. (2014) 30:37–40. doi: 10.13412/j.cnki.zyyl.2014.01.013

[101] Cheng F Zhao M Wang Q Xiong H Yu K Chen C . FOXO1-NMNAT3 axis dysregulation promotes doxorubicin cardiotoxicity: NAD^+^ replenishment as a redox-targeted antioxidant therapy. Redox Rep Commun Free Radic Res. (2025) 30:2565033. doi: 10.1080/13510002.2025.2565033 41021886 PMC12481541

[102] Zhou Y Zhang H Huang Y Wu S Liu Z. Tanshinone IIA regulates expression of glucose transporter 1 via activation of the HIF-1α signaling pathway. Mol Med Rep. (2022) 26:328. doi: 10.3892/mmr.2022.12844 36069225 PMC9727584

[103] Baburina Y Lomovsky A Lomovskaya Y Sotnikov R Sotnikova L Krestinina O. Mitochondrial protection by astaxanthin reduces toxicity caused by H_2_O_2_ and doxorubicin in human cardiomyocytes. Cells. (2025) 14:1772. doi: 10.3390/cells14221772 41294824 PMC12650927

[104] Wu S Lu D Gajendran B Hu Q Zhang J Wang S . Tanshinone IIA ameliorates experimental diabetic cardiomyopathy by inhibiting endoplasmic reticulum stress in cardiomyocytes via SIRT1. Phytother Res. (2023) 37:3543–58. doi: 10.1002/ptr.7831 37128721

[105] Hou J Ren J Abbas S Song Y Huang H Cao M . Targeting the gut-heart axis: dextran from gut-derived *Weissella cibaria* alleviates doxorubicin-induced cardiotoxicity by attenuating low-grade endotoxemia. J Agric Food Chem. (2025) 73:33084. doi: 10.1021/acs.jafc.5c08925 41403204

[106] Zhu T Chen J Zhang M Tang Z Tong J Hao X . Tanshinone IIA exerts cardioprotective effects through improving gut-brain axis post-myocardial infarction. Cardiovasc Toxicol. (2024) 24:1317–34. doi: 10.1007/s12012-024-09928-4 39377990 PMC11564317

[107] Dai Q Pan Y Zhu X Chen M Xie L Zhu Y . Network pharmacology along with molecular docking to explore the mechanism of Danshen injection against anthracycline-induced cardiotoxicity and transcriptome validation. Curr Pharm Des. (2024) 30:952–67. doi: 10.2174/0113816128289845240305070522 38482629

[108] Kong J Li T Feng X. Clinical efficacy research on Tongluo Ningxin decoction in the prevention of cardiac toxicity caused by anthracyclines. Chin J Mod Drug Appl. (2018) 12:1–4. doi: 10.14164/j.cnki.cn11-5581/r.2018.21.001

[109] Peng L Fan M Li J Chen W. Evidence quality assessment of sodium Tanshinone IIA sulfonate injection intervention coronary heart disease angina pectoris: an overview of systematic reviews and meta-analyses. Medicine. (2023) 102:e35509. doi: 10.1097/MD.0000000000035509 37933033 PMC10627678

[110] Wu X Fan M Wei S Guo D. The efficacy and safety of sodium tanshinone IIA sulfonate injection in the treatment of unstable angina pectoris: a systematic review and meta-analysis. PLoS ONE. (2023) 18:e0290841. doi: 10.1371/journal.pone.0290841 37651454 PMC10470971

[111] Li S Jiao Y Wang H Shang Q Lu F Huang L . Sodium Tanshinone IIA sulfate adjunct therapy reduces high-sensitivity C-reactive protein level in coronary artery disease patients: a randomized controlled trial. Sci Rep. (2017) 7:17451. doi: 10.1038/s41598-017-16980-4 29234038 PMC5727111

[112] Kuriakose RK Kukreja RC Xi L. Potential therapeutic strategies for hypertension-exacerbated cardiotoxicity of anticancer drugs. Oxid Med Cell Longev. (2016) 2016:8139861. doi: 10.1155/2016/8139861 27829985 PMC5086499

[113] Li S Li W Cheng M Wang X Chen W. Prevention and treatment of anthracycline-induced cardiotoxicity: a systematic review and network meta-analysis of randomized controlled trials. Cardio-Oncology. (2025) 11:66. doi: 10.1186/s40959-025-00360-3 40640889 PMC12243438

[114] Wang T Wang C Wu Q Zheng K Chen J Lan Y . Evaluation of Tanshinone IIA developmental toxicity in zebrafish embryos. Molecules. (2017) 22:660. doi: 10.3390/molecules22040660 28430131 PMC6154573

[115] Tan X Cheng X Yang Y Yan L Gu J Li H . Tanshinone II-A sodium sulfonate (DS-201) enhances human BKCa channel activity by selectively targeting the pore-forming α subunit. Acta Pharmacol Sin. (2014) 35:1351–63. doi: 10.1038/aps.2014.85 25345746 PMC4220078

[116] Yang L Huang X Wang Z Guo Z Ma C Dong L . Research progress on the pharmacological properties of active ingredients from *Salvia miltiorrhiza*: a review. Phytomedicine. (2025) 148:157272. doi: 10.1016/j.phymed.2025.157272 40997663

[117] Li C Zheng Y Niu D Zhu R Yan X Qu H . Effects of traditional Chinese medicine injections for anthracyclines-induced cardiotoxicity: an overview of systematic reviews and meta-analyses. Integr Cancer Ther. (2023) 22:15347354231164753. doi: 10.1177/15347354231164753 37057304 PMC10108409

[118] Yang Q Xiang X Wang H Liao Y Li X. Oral natural material hydrogels: a new strategy for enhancing oral drug delivery efficiency. J Biomater Sci Polym Ed. (2025) 36:2758. doi: 10.1080/09205063.2025.2509028 40418586

[119] Szponar J Ciechanski E Ciechanska M Dudka J Mandziuk S. Evolution of theories on doxorubicin-induced late cardiotoxicity-role of topoisomerase. Int J Mol Sci. (2024) 25:13567. doi: 10.3390/ijms252413567 39769331 PMC11678604

[120] Deng S Yan T Jendrny C Nemecek A Vincetic M Gödtel-Armbrust U . Dexrazoxane may prevent doxorubicin-induced DNA damage via depleting both topoisomerase II isoforms. BMC Cancer. (2014) 14:842. doi: 10.1186/1471-2407-14-842 25406834 PMC4242484

[121] Ma Z Wu Y Xu J Cao H Du M Jiang H . Sodium Tanshinone IIA sulfonate ameliorates oxygen-glucose deprivation/reoxygenation-induced neuronal injury via protection of mitochondria and promotion of autophagy. Neurochem Res. (2023) 48:3378–90. doi: 10.1007/s11064-023-03985-x 37436612

[122] Wu P Chen L Xu Y-X Ren Y-Y Dong X-M Fan J-X . Decoding herbal medicine: AI-powered omics and network pharmacology. Phytomedicine. (2025) 148:157453. doi: 10.1016/j.phymed.2025.157453 41205526

[123] Jirkovský E Jirkovská A Bureš J Chládek J Lenčová O Stariat J . Pharmacokinetics of the cardioprotective drug dexrazoxane and its active metabolite ADR-925 with focus on cardiomyocytes and the heart. J Pharmacol Exp Ther. (2018) 364:433–46. doi: 10.1124/jpet.117.244848 29273587

[124] Hasinoff BB Patel D Wu X. The role of topoisomerase IIβ in the mechanisms of action of the doxorubicin cardioprotective agent dexrazoxane. Cardiovasc Toxicol. (2020) 20:312–20. doi: 10.1007/s12012-019-09554-5 31773441

[125] Zhang Y Ahmad KA Khan FU Yan S Ihsan AU Ding Q. Chitosan oligosaccharides prevent doxorubicin-induced oxidative stress and cardiac apoptosis through activating p38 and JNK MAPK mediated Nrf2/ARE pathway. Chem Biol Interact. (2019) 305:54–65. doi: 10.1016/j.cbi.2019.03.027 30928397

[126] Yu X Ruan Y Huang X Dou L Lan M Cui J . Dexrazoxane ameliorates doxorubicin-induced cardiotoxicity by inhibiting both apoptosis and necroptosis in cardiomyocytes. Biochem Biophys Res Commun. (2020) 523:140–6. doi: 10.1016/j.bbrc.2019.12.027 31837803

[127] Tebbi CK London WB Friedman D Villaluna D De Alarcon PA Constine LS . Dexrazoxane-associated risk for acute myeloid leukemia/myelodysplastic syndrome and other secondary malignancies in pediatric Hodgkin's disease. J Clin Oncol. (2007) 25:493–500. doi: 10.1200/JCO.2005.02.3879 17290056

[128] Zhu P-C Shen J Qian R-Y Xu J Liu C Hu W-M . Effect of Tanshinone IIA for myocardial ischemia/reperfusion injury in animal model: preclinical evidence and possible mechanisms. Front Pharmacol. (2023) 14:1165212. doi: 10.3389/fphar.2023.1165212 37261285 PMC10228700

[129] Yang C Mu Y Li S Zhang Y Liu X Li J. Tanshinone IIA: a Chinese herbal ingredient for the treatment of atherosclerosis. Front. Pharmacol. (2023) 14: 1321880. doi: 10.3389/fphar.2023.1321880 38108067 PMC10722201

[130] Gao S Liu Z Li H Little PJ Liu P Xu S. Cardiovascular actions and therapeutic potential of Tanshinone IIA. Atherosclerosis. (2012) 220:3–10. doi: 10.1016/j.atherosclerosis.2011.06.041 21774934

[131] Huo M Tang Z Wang L Zhang L Guo H Chen Y . Magnesium hexacyanoferrate nanocatalysts attenuate chemodrug-induced cardiotoxicity through an anti-apoptosis mechanism driven by modulation of ferrous iron. Nat Commun. (2022) 13:7778. doi: 10.1038/s41467-022-35503-y 36522337 PMC9755285

[132] Langer SW. Dexrazoxane for the treatment of chemotherapy-related side effects. Cancer Manag Res. (2014) 6:357–63. doi: 10.2147/CMAR.S47238 25246808 PMC4168851

[133] Vogel CL Gorowski E Davila E Eisenberger M Kosinski J Agarwal RP . Phase I clinical trial and pharmacokinetics of weekly ICRF-187 (NSC 169780) infusion in patients with solid tumors. Invest New Drugs. (1987) 5:187–98. doi: 10.1007/BF00203545 3115912

[134] Liu HC Liu HH. Adverse reactions of tanshinone II(A) sodium sulfonate injection in treating 18 cases: an analysis of clinical features. Zhongguo Zhong Xi Yi Jie He Za Zhi. (2013) 33:1287–89. 24273991

[135] Jiang S Li H Zhang L Mu W Zhang Y Chen T . Generic Diagramming Platform (GDP): a comprehensive database of high-quality biomedical graphics. Nucleic Acids Res (2025) 53:D1670–6. doi: 10.1093/nar/gkae973 39470721 PMC11701665

